# SeqVItA: Sequence Variant Identification and Annotation Platform for Next Generation Sequencing Data

**DOI:** 10.3389/fgene.2018.00537

**Published:** 2018-11-14

**Authors:** Prashanthi Dharanipragada, Sampreeth Reddy Seelam, Nita Parekh

**Affiliations:** Center for Computational Natural Science and Bioinformatics, International Institute of Information Technology, Hyderabad, India

**Keywords:** SNPs, INDELs, sequence variants, NGS, annotation, personalized medicine, platform

## Abstract

The current trend in clinical data analysis is to understand how individuals respond to therapies and drug interactions based on their genetic makeup. This has led to a paradigm shift in healthcare; caring for patients is now 99% information and 1% intervention. Reducing costs of next generation sequencing (NGS) technologies has made it possible to take genetic profiling to the clinical setting. This requires not just fast and accurate algorithms for variant detection, but also a knowledge-base for variant annotation and prioritization to facilitate tailored therapeutics based on an individual's genetic profile. Here we show that it is possible to provide a fast and easy access to all possible information about a variant and its impact on the gene, its protein product, associated pathways and drug-variant interactions by integrating previously reported knowledge from various databases. With this objective, we have developed a pipeline, Sequence Variants Identification and Annotation (SeqVItA) that provides end-to-end solution for small sequence variants detection, annotation and prioritization on a single platform. Parallelization of the variant detection step and with numerous resources incorporated to infer functional impact, clinical relevance and drug-variant associations, SeqVItA will benefit the clinical and research communities alike. Its open-source platform and modular framework allows for easy customization of the workflow depending on the data type (single, paired, or pooled samples), variant type (germline and somatic), and variant annotation and prioritization. Performance comparison of SeqVItA on simulated data and detection, interpretation and analysis of somatic variants on real data (24 liver cancer patients) is carried out. We demonstrate the efficacy of annotation module in facilitating personalized medicine based on patient's mutational landscape. SeqVItA is freely available at https://bioinf.iiit.ac.in/seqvita.

## Background

Precision medicine is an emerging approach for risk assessment to diagnosis, disease prognosis, and treatment that considers individual genetic variability into account. It requires analyzing multiple genes quickly and sensitively from small quantities of sample, which is now possible with the advent of next generation sequencing (NGS) techniques. Diagnostic testing of Mendelian and heredity disorders and risk screening of heredity cancers are a few well-established clinical applications of NGS techniques. Targeted sequencing (TS) is widely used in the form of gene panels, e.g., hotspot panels (either clinically actionable or with diagnostic/prognostic significance), actionable gene panels (includes all exons of targeted genes), and disease-focused gene panels. With the decrease in the cost of sequencing, whole genome (WGS), and whole exome sequencing (WES) are slowly emerging in biomedical studies and medical practices. The major advantage with WGS/WES approaches is that one can identify mutations not previously reported and in non-coding regions (with WGS), which may be specific to the individual. Thus, the revolution of high-throughput sequencing technologies has made it possible to carry out genome-wide analysis of somatic mutations in population-scale cancer cohorts.

Major steps involved in the prediction of sequence variants in clinical NGS data are (1) Data pre-processing, (2) Read alignment, (3) Variant calling, and (4) Variant annotation and prioritization. Data pre-processing involves removing low quality reads, adapter sequences and contamination. It is a key step in any NGS data analysis and has a direct effect on the downstream analysis if not performed properly. A number of data quality check, filtering and trimming tools are available (both standalone and web-based) (Del Fabbro et al., 2013; Bao et al., 2014). A major limitation with some of these tools is that they are unable to handle very large datasets. For example, PRINSEQ (Schmieder and Edwards, 2011) works efficiently for a single sample, while NGS QC Toolkit (Patel and Jain, 2012) and FastQC (Andrews, 2010) can handle small number of samples simultaneously; however, the performance is very poor in terms of runtime and memory usage for large datasets. Raspberry (Katta et al., 2015) is able to efficiently perform batch processing on large number of samples in parallel and FaQCs (Lo and Chain, 2014) enables quality check, trimming and filtering of low-quality reads in large samples quickly through multi-threading.

Alignment of pre-processed short sequence reads to a reference genome is the next step which requires large computation space and is highly time-consuming. BWA (Li and Durbin, 2009) and Bowtie2 (Langmead and Salzberg, 2012) are some of the fastest aligners available that use “indexing” strategies (compression heuristics), resulting in optimal alignment of reads. Most of the existing aligners have a provision to map the reads using multiple cores/CPUs (parallel processing). Standalone tools such as NGS-QCbox (Katta et al., 2015), cloud-based aligners such as CloudBurst (Schatz, 2009), Galaxy CloudMan (Afgan et al., 2010), and Crossbow (Gurtowski et al., 2012) carry out read mapping much efficiently using big data infrastructure.

Because of large space-time resources required for the above two steps in NGS data analysis, most variant callers take either the alignment file in BAM format or the VCF format as input. Variants are predicted by comparing each position with the standard reference sequence and applying certain statistical models/heuristics to improve the reliability of predictions. Some popular tools for detection of SNVs and small INDELs in large NGS datasets are summarized in Table 1, namely, GATK (based on Hadoop-MapReduce framework) (McKenna et al., 2010; Cibulskis et al., 2013), MAFsnp (Hu et al., 2015), and SNVSniffer (Liu et al., 2016). Sequence variants are classified as germline, somatic, or loss-of-heterozygosity (LOH) by the variant callers. Germline variants are usually identified from a single, paired, family-pedigree sequences or population cohorts, while a matched normal is required for the prediction of somatic and LOH variants. Each of these tools have their inherent strengths and limitations and none of these can reliably detect all types and sizes of sequence variants (Yi et al., 2014; Xu, 2018).

**Table 1:** Feature comparison of SeqVItA with various popular and recent tools for sequence variant calling, annotation and prioritization in NGS data.

	**Variant Callers**						**Annotation**			**Integrated Platforms**				
	**GATK**	**BCFtools**	**VarScan2**	**DeepVariant**	**SNVsniffer**	**MAFsnp**	**ANNOVAR**	**CADD**	**FCVPP2**	**CoVaCs**	**RAREVATOR**	**Canary**	**Nimbus**	**SeqVItA**
**DATA INPUT**														
Single	✓	✓	✓		✓	✓	✓	✓	✓	✓	✓	✓	✓	✓
Paired (case-control)	✓	–	✓	–	✓	–	–	–	–	-	✓	✓	✓	✓
Multiple	✓	✓	✓	–		✓	–	–	–	✓	–	–	–	✓
Read alignment	–	–	–	–	–	–	–	–	–	✓	–	✓	✓	–
Preprocessing post-alignment	✓	✓	–	✓	–	–	–	–	–	✓	–	–	–	✓
**VARIANT CALLING**														
Germline	✓	✓	✓	✓	✓	✓	–	–	–	✓	✓	✓	✓	✓
Somatic	✓	–	✓	–	✓	–	–	–	–	–	✓	✓	✓	✓
Multi-allelic F	✓	–	–	–	–	✓	–	–	–	–	–	–	–	✓
Parallelization	✓	✓	✓	✓	–	–	–	–	–	✓	–	–	–	✓
**ANNOTATION**														
Gene-locus	–	–	–	–	–	–	✓	✓	✓	✓	✓	✓	✓	✓
Conservation score	–	–	–	–	–	–	✓	✓	✓	✓	–	✓	✓	✓
Population alleles	–	–	–	–	–	–	✓	✓	✓	✓	✓	–	✓	–
Clinical associations	–	–	–	–	–	–	✓	✓	✓	–	✓	✓	✓	✓
Drug associations	–	–	–	–	–	–	–	–	–	–	–	–	–	✓
Prioritization	–	–	–	–	–	–	–	✓	✓	✓	✓	✓	–	✓
Reference	McKenna et al., 2010	Li, 2011	Koboldt et al., 2012	Poplin et al., 2017	Liu et al., 2016	Hu et al., 2015	Wang et al., 2010	Kircher et al., 2014	Kumar et al., 2018	Chiara et al., 2018	Magi et al., 2015	Doig et al., 2017	Brouwer et al., 2018

Variant calling in WGS (WES) data result in millions (hundreds) of variants, most of which may not contribute to any phenotypic condition. Identifying a small subset of functional variants that are directly involved in a pathway/disease is desirable. The first step in variant prioritization is annotation. This includes identifying the functional impact of the variant on the gene/protein product, its frequency in the population, disease association and variant-drug association. Some popular variant annotation tools are listed in Table 1. The most common annotation feature across these tools is gene-locus annotation, i.e., position of variant on the gene locus (exon/intron/5′/3′ UTR/promoter). Features such as phylogenetic conservation scores [e.g., SIFT (Ng and Henikoff, 2003) and PolyPhen2 (Adzhubei et al., 2013)], help in distinguishing the impact of the variants—from damaging to tolerated missense variants, while population allele frequencies [dbSNP (Pagès, 2017), 1000 Genome Project (1000 Genomes Project Consortium et al., 2015) and gnomAD (Lek et al., 2016)] help in filtering common variations. Known variant-disease associations can be obtained from databases such as OMIM (Hamosh et al., 2002), ClinVar (Landrum et al., 2014), and COSMIC (Bamford et al., 2004) and can help in understanding the role of variants in disease prevalence and mode of inheritance. Another important class of annotation is variant-drug association that enables us to interpret efficacy of the drug in the presence of a particular variant, e.g., PharmGKB (Gong et al., 2008). Since not all variants in the genic region are deleterious, it is necessary to prioritize the predicted variants. Methods proposed vary from computing simple cumulative scores to statistical measures based on available information for the variant in various resources. Thus, annotation and prioritization of variants is prerequisite in personalized therapy.

It may be noted from Table 1 that majority of the existing tools are either only variant callers or annotation provider, with a limited few providing both the features. Further, variant callers may be limited by data types (single, paired, or pooled samples), aligner outputs, and computational efficiency. One of the most widely used annotation tool, ANNOVAR, requires input in a specific format that is not supported by most variant callers, making it difficult to use. There are numerous papers that propose pipeline for disease-specific variant detection and annotation (Alexander et al., 2016; Krupp et al., 2017; Mathur et al., 2018). These are useful in guiding the experimentalist but require the user to download and install all the tools and dependencies. To address some of these issues we have developed an open-source, command-line driven pipeline, SeqVItA. On a single platform all the data types and output formats are supported and parallelization of the variant calling step makes it easy to handle large datasets. A detailed annotation is provided for both coding and non-coding variants and based on their functional impact a score is given which is used for prioritizing the variant. Here we show how the annotation module of SeqVItA can assist in making a suitable treatment plan based on an individual's mutational profile. The efficacy of both the variant calling and annotation modules are discussed.

## Methods

### Overview of SeqVitA

**Figure 1:**
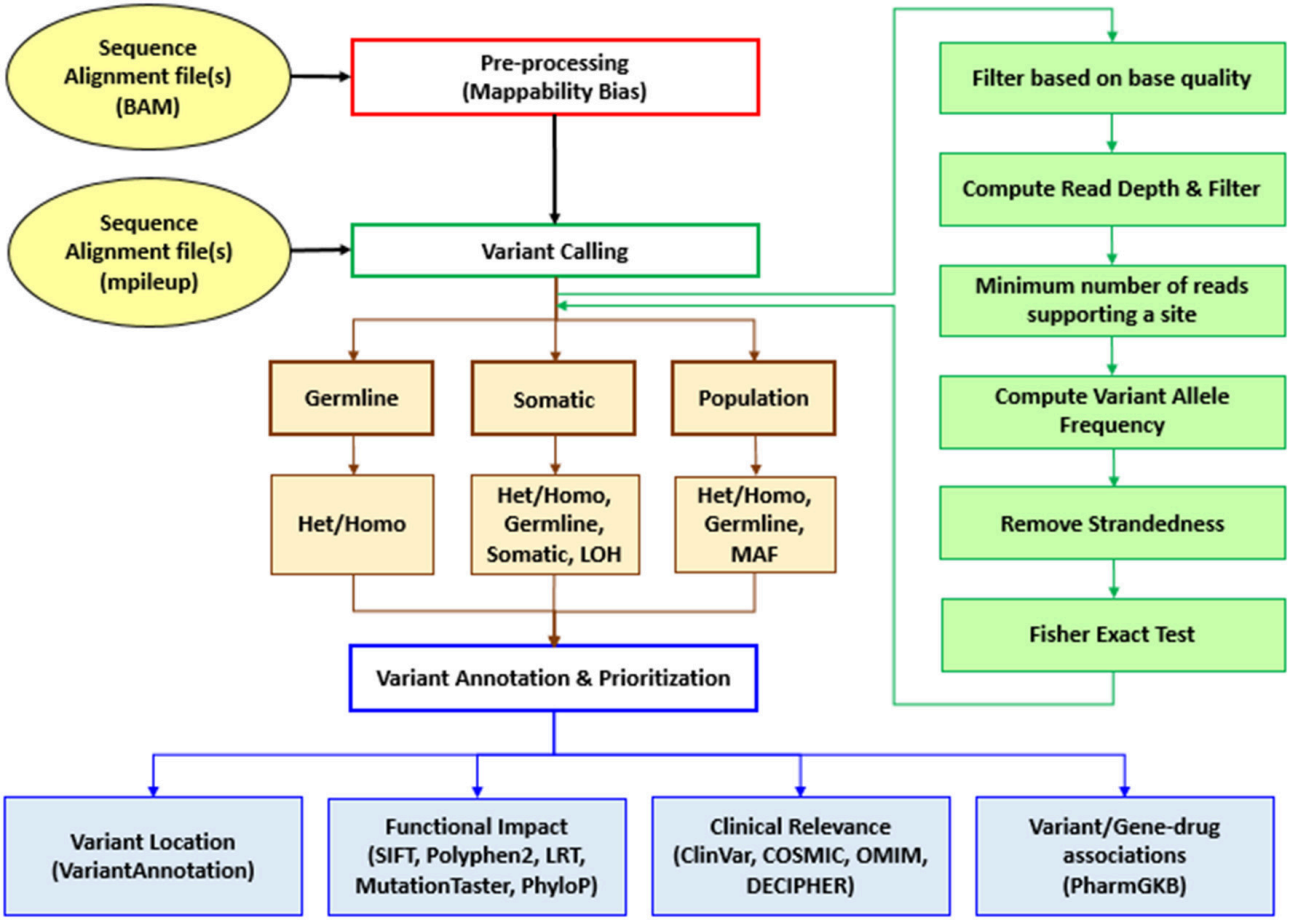
Workflow of SeqVItA for identification, annotation and prioritization of sequence variants in WGS, WES, or TS data. Het, Heterozygous; Homo, Homozygous; LOH, Loss of heterozygosity; MAF, Minor allele frequency.

Sequence Variants Identification and Annotation (SeqVItA) is an open-source platform for the prediction and annotation of SNVs and small INDELs in whole genome (WGS), whole exome (WES), and targeted (TS) next generation sequencing (NGS) data. It can handle single or multiple input files simultaneously in either BAM or mpileup format. In SeqVItA, one can detect germline mutations in single samples and population datasets (wherein the frequency of a minor allele is also computed). For the detection of somatic variations in cancer data, it uses paired tumor-control samples. It is built on a modular framework and the workflow of SeqVItA is shown in Figure 1. It consists of three major modules, *viz*., (i) pre-processing, (ii) variant calling, and (iii) variant annotation and prioritization. The variant calling module consists of three sub-modules: germline, somatic, and population to handle different type of data analyses. SeqVItA is freely available at https://bioinf.iiit.ac.in/seqvita.

#### Implementation

SeqVItA follows a heuristic/statistic approach similar to VarScan2 (Koboldt et al., 2012) for variant detection. It is implemented in a combination of programming languages (C++, R, and Bash). The input to SeqVItA is aligned file in either BAM or mpileup format and the computationally intensive variant calling step is parallelized using OpenMP. It involves filtering the variants based on predefined criteria for read depth, base quality, variant allele frequency and statistical significance. Additionally, SeqVItA provides annotation and prioritization to the predicted variants for assessing the biological significance of the variants called. This module can also be independently accessed by the researchers for variants predicted from any other tool. Various steps, parameters and their default values considered in the prediction of SNVs and INDELs in SeqVItA are summarized in Table 2.

**Table 2:** Various steps and corresponding parameters in the detection of SNVs and INDELs in SeqVItA are summarized.

**Steps**	**Description**	**Key Parameter**	**Value**
Pre-processing (aligned file in BAM format)	Mapping quality correction using Equation (1)	–Mqcorr	0
	Filter reads based on mapping quality cut-off	–Mqread	20
Variant Calling	Base quality Cut-off (Phred score)	–Qbase	15
	Read depth at a site ≥ cut-off, site is considered for variant calling	–RD_th	10
	If no. of reads supporting alternate allele ≥ cut-off, site is considered for variant calling	–VAR_th	2
	Compute variant allele frequency (VAF) for variant calling	–VAF_th	0.20
	Check for strand bias (Discard if ≥ 90% and ≤ 10% support from same strand)	–Strand_Bias	1
	*p*-value cut-off (FET*) for calling variants	–p-value	0.01
	For germline SVs, if VAF > cut-off, variant is homozygous, else heterozygous	–VAF_homo	0.75
	*p*-value cut-off (FET*) for calling somatic, LOH variants	–somatic-p-value	0.05
Variant annotation	Drug association based on gene/variant in PharmGKB	-d	–

* *FET: Fisher Exact test*.

### Pre-processing

This step is used only when the input is alignment file in BAM format. Variant calling is very sensitive to the quality of alignment, as a wrong alignment of reads may lead to false positives. Hence, it is extremely important to ensure that the reads are uniquely mapped. To handle the mappability issue, mpileup function in Samtools has been incorporated for recalibrating and filtering of low mapping quality reads. Mapping quality of the reads is recalibrated using the expression:

(1) $$\begin{array}{r} Mq^{\prime }=\;\sqrt{\frac{Int-Mq}{Int}}\;\times Int \end{array}$$

where Int is a user defined integer and Mq is the phred-scaled probability of a read being misaligned. Default *Mqcorr* = 0 implies no mappability correction (*Mqcorr* = 50 is recommended for the alignment files generated using BWA or Bowtie2). The recalibrated file is generated in an mpileup format for further analysis. If the alignment has been obtained using only uniquely mapped reads by an aligner, one may skip this step. This is followed by filtering of low mapping quality reads (≤ mapping quality), which is user-defined depending on the aligner used. If the input to SeqVItA is in mpileup format, this step is not required.

### Variant calling

In this step, depending on the type of data, the user may choose one of the three modules for small sequence variant detection: ***germline*** module (single sample), ***somatic*** module (paired tumor-control samples) and ***population*** module (multiple samples). Using ***germline*** module one may identify SNVs, INDELs or both simultaneously in a single sample, while identification of somatic, LOH and germline sequence variants (SNVs and/or INDELs) is carried out in ***somatic*** module for paired tumor-control samples. SeqVItA also handles genotyping in population data using ***population*** module that takes multiple sample files simultaneously as input and computes frequency of minor allele in the dataset. The input is alignment file in mpileup format and the output is in variant call format (VCF, v4.1). The details of each of these modules are discussed below.

#### Variant calling in single sample

The input to this module is mpileup format file, shown in Supplementary Figure S1. It is a tab-separated text file that contains information about the reads aligned to reference genome. First four columns give the chromosome number, position in reference genome, nucleotide present at the location in reference genome and number of reads aligned at that position, respectively. In the 5th column, base aligned at the position in the sample: “,” or “.” if the base at the location is the same as that in reference, characters (“A”, “G”, “T”, or “C”) for substitution (SNV) and “+” or “−” followed by an integer representing insertion/deletion of integer length at the position. The 6th column gives ASCII encoded base qualities of the base in the reads.

For variant calling, each line in the mpileup file is parsed and the coverage at each location is computed by considering only those reads that have base quality ≥ Qbase (= 15, default). For *N* samples, coverage and quality of the bases is be obtained from 4+3(*m*−1) and 6+3(*m*−1) columns respectively, for sample *m* = 1, 2, …, *N*. If the number of high quality reads ≥ minimum coverage threshold, RD_th (= 10, default) at a location, then the site is considered for further analysis. To make a variant call at a position, SeqVItA checks whether minimum number of reads supporting the variant, VAR_th (= 2, default) is satisfied. Variant allele frequency (VAF) is then computed by taking the ratio of the number of non-reference alleles to the total number of reads at the locus. Further, if VAF ≥ VAF_th, it is checked for strandedness. The strand bias is identified if majority of reads supporting the variant (>90%) belong to the same strand (forward or reverse). The strand bias may occur due to erroneous PCR duplication and the variants called at such locations are discarded.

Variants passing the above filtering criteria are considered for Genotype call, Genotype Quality, and *p*-value calculation to assess the significance of the variant called. This is done by using one-tailed Fisher exact test under the null hypothesis that the variation observed is due to sequencing error. For this, number of reads supporting the variant is compared to the expected distribution of reads for a non-variant position based on sequencing error alone. Suppose, N(obs, ref) and N(obs, var) denote number of reads supporting the reference and variant respectively, with the total coverage at a location defined as N(obs) = N(obs, ref) + N(obs, var). Considering sequencing error to be 0.001, expected number of reads supporting the variant allele due to sequencing error is N(exp, var) = N(obs) × 0.001. The expected number of reads supporting the reference in this case is N(exp, ref) = N(obs) − N(exp, var). If the observed number of reads supporting the variant, N(obs, var) > N(exp, var), the site is assumed to be a true variant, else a sequencing error. Representing these four quantities in a 2 × 2 contingency table (Table 3), the probability of obtaining the observed data is given by

(2) $$\begin{array}{r} p=\frac{{C_{N\left(obs,\;var\right)}^{N\left(var\right)}\times \;C}_{N\left(obs,\;ref\right)}^{N\left(ref\right)}}{C_{N\left(Obs\right)}^{N}} \end{array}$$

where, N(var) and N(ref) represent row total for variant and reference alleles, respectively. The probability for all values of N(obs,var) is computed by considering all possible tables obtained by reducing the least value in the table (N(exp, var)) to zero while keeping the row total and column total constant. The *p*-value is then defined as the sum of all the hypergeometric probabilities of these contingency tables. If *p*-value is less than the cutoff (= 0.05, default), the site is said to be a true variant. The Genotype Quality is then given by:

(3) $$\begin{array}{r} \text{Genotype Quality}=-10\text{log }\left(\text{p}\text{-value}\right) \end{array}$$

**Table 3:** The 2 × 2 contingency table for computing the *p*-value using Fisher exact test.

**Reads supporting**	**Observed**	**Expected**	**Row Total**
Variant	N(obs, var)	N(exp, var)	N(var)
Reference	N(obs, ref)	N(exp, ref)	N(ref)
Column total	N(obs)	N(exp)	N

Once the variants are identified, a final filter is applied to categorize variants as heterozygous or homozygous. If the variant allele frequency at a location is greater than the user-defined homozygous frequency cutoff, VAF_homo (= 0.75, default), the site is said to be Homozygous (HOM = 1), else Heterozygous (HET = 1).

#### Variant calling in case-control samples

For identifying somatic, germline and LOH sequence variants, tumor samples along with a matched control sample paired samples (either as two separate alignment files in BAM format, or a single mpileup file for both normal and tumor alignment files) is given to the ***somatic*** module. In this case, the first three columns contain information on chromosome number, position and nucleotide present at the location, columns 4th, 5th, and 6th contain information regarding read depth, reference/variant base, and base quality for sample 1 and corresponding information for sample 2 is given in columns 7th, 8th, and 9th in the mpileup file. As in ***germline*** module, in this case also each location is assessed based on the coverage requirement. The *p*-value calculation is then carried out to determine the genotype in the two samples independently if the filtering criteria are satisfied. If the filtering criteria are not met for any one of the case/control samples, the site is said to be homozygous (reference). If the number of reads supporting a variant allele is >75% (VAF_homo), the variant is called homozygous, else heterozygous (as in ***germline*** module). In case of multiple variant alleles observed at a location, the variant alleles with higher read count, followed by base quality are reported. Positions at which one or both the samples have a variant and the genotypes do not match, a comparison between the tumor and control samples is carried out using one-tailed Fisher exact test in 2 × 2 contingency tables (Table 4) to compute an additional *p*-value called somatic *p*-value (Supplementary Figure S2). This helps in distinguishing the somatic and LOH variants from germline mutations, as shown below.

**Table 4:** The 2 × 2 contingency table for computing *p*-value using Fisher exact test to predict somatic, germline and LOH mutations.

	**Reference**	**Variant**	**Row total**
Tumor	N(tum, ref)	N(tum, var)	N(tum)
Normal	N(nor, ref)	N(nor, var)	N(nor)
Column total	N(ref)	N(var)	N

*Total number of reads, N = Nref + Nvar = Ntum + Nnor, where Nref and Nvar correspond to the number of reads supporting the reference and variant, respectively and Ntum and Nnor correspond to the number of reads in tumor and normal samples, respectively*.

Here, number of reads supporting the reference allele N(tum, ref) and a variant allele N(tum, var) in the tumor sample are compared to that observed in the control sample, N(nor, ref) and N(nor, var). Under the null hypothesis that reference alleles and variants alleles are independently distributed in tumor and normal samples, the probability of observing ***x*** reference alleles in the tumor sample is given by

(4) $$\begin{array}{r} p=\frac{C_{x}^{N\left(tum\right)}\times \;C_{N\left(ref\right)-x}^{N\left(nor\right)}}{C_{N\left(var\right)}^{N}} \end{array}$$

The probability of ***x*** reference alleles in the tumor sample is computed by considering all possible tables obtained by reducing the least value in the table to zero while keeping the row totals and column totals constant. The somatic *p*-value is then defined as the sum of all the hypergeometric probabilities of these contingency tables. If this *p*-value ≤ somatic-*p*-value threshold (= 0.05, default) and the normal matches the reference allele, the base is classified as “Somatic” and if the normal is heterozygous, the base is classified as “LOH.” If the somatic *p*-value for a base is smaller than the threshold cut-off (0.05) and the normal base do not meet the above criteria, the base is classified as “Unknown.” Sequence variants where the allele is homozygous in normal, and homozygous-reference or heterozygous in the tumor are grouped under “Unknown” category. In case somatic *p*-value is greater than the threshold cut-off or the genotypes in the case-control samples match, the variant *p*-value (VPV) is computed by combining tumor and normal read counts for each allele under the null hypothesis that the site is non-variant (i.e., variation observed is due to sequencing error) and the base is reported as “Germline.” Based on the variant allele frequency (VAF) and somatic *p*-values (one-tailed Fisher Exact test), a somatic variant is reported as “High” priority if VAF ≥ 10% in tumor, < 5% in normal and *p* < 0.07, and LOH is reported as “High” priority if VAF ≥ 10% in normal and *p* < 0.07. Germline variants are described as “High” priority if VAF ≥ 10% in both tumor and normal samples (Supplementary Figure S2). Any sequence variant not meeting these criteria is assigned “Low” priority. Thus, the user may filter out high confidence sequence variants. For this module, SeqVItA generates four VCF files using the function ***splitVCF*** corresponding to Somatic, Germline, LOH and Unknown sequence variants.

#### Variant calling in large population samples

Apart from detecting variants in single and paired samples, SeqVItA also handles population data for genotyping in disease cohort studies. In this case, the input is multiple alignment files (BAM format) or a single mpileup file containing information of multiple samples. The sequence variant detection and *p*-value computation are carried out individually for each sample using one-tailed Fisher Exact Test (similar to ***germline*** module). A variant site may have more than one allele identified in multiple samples and these alleles are reported under “ALT” column, as shown in Supplementary Figure S3. Samples with no sequence variant are represented by 0/0 and 0/1 if the sample contains one variant allele. This is reported in the respective sample information columns. In case more than one alternate allele is identified in any sample, then it is represented as 0/2 under that sample and both the alleles are reported in the “ALT” column (comma separated) in VCF file. Minor allele frequency (MAF), defined as the frequency of second most common allele identified in the given population dataset, is also computed for each variant. Based on MAF value, user may categorize the predicted sequence variant as “rare” (MAF < 0.01) or “common” (MAF > 0.01). MAF is reported as percentage frequency of the allele in the “INFO” column, MAF = NA implies no minor allele is identified.

### Parallelization of the variant calling step

Screening for variants at every position of the 3 billion bases in human genome sequentially is very time-consuming. To improve computational efficiency, we have parallelized the variant calling step in SeqVItA using OpenMP that supports shared memory multi-processing. In this parallel computing platform, variant calling is carried out simultaneously for “*n*” positions by using “*n*” threads in which each thread shares the same memory address space resulting in a faster communication. Compared to other existing frameworks such as OpenMPI or MapReduce, OpenMP is chosen for its efficiency in carrying out computationally-intensive tasks (Kang et al., 2015).

### Annotation

Typically, small sequence variants (SNVs and INDELs) range from thousands to millions in number in a genome. However, not all variants are likely to be associated to a phenotype. Identifying a small subset of functional variants that are directly involved in a pathway/disease is a major challenge in research. Though there exists a number of variant callers, in our knowledge very few provide any annotation/prioritization to the called variants. To address this problem, we have developed the annotation module that extracts information from numerous resources to assess the significance of the predicted variants. Thus, using this module, the user may filter out non-significant variants for further analysis. This module can also be used independently by the user to annotate variants obtained from any other sequence variant detection tool in VCF format. Most trivial way to prioritize the sequence variants is by statistical significance given by *p*-value (Fisher's exact test). In SeqVItA, the *p*-value is provided in the INFO column of the VCF file and the user may sort and filter the variants based on this column. Though *p*-value gives the statistical significance, it does not tell us anything about the biological significance of the variant. For evaluating the biological significance, we provide four levels of information. First level of screening is based on the location of the variant: whether it is intergenic or intragenic. R package “VariantAnnotation” (Obenchain et al., 2014) integrated in SeqVItA provides gene-centric annotation to the variant, e.g., whether the variant lies in the coding region (synonymous, non-synonymous, frameshift, amino acid change), intron, 5′/3′ UTR, splice site, promoter, or intergenic region. For intergenic variants, gene preceding or following the variant is provided. Known variants from dbSNP are reported using R package “SNPlocs.Hsapiens.dbSNP144.GRCh37” (Pagès, 2017). In SeqVItA, functional annotation of the predicted variant is provided at three levels: (i) functional impact of the mutation, (ii) clinical association, and (iii) variant/gene-drug association. For functional impact of the variant, R package “rfPred” (Jabot-Hanin et al., 2016) is used to extract SIFT (Ng and Henikoff, 2003), Polyphen2 (Adzhubei et al., 2013) MutationTaster (Schwarz et al., 2010), PhyloP (Pollard et al., 2010) and likelihood ratio test (LRT) (Chun and Fay, 2009) scores from the database of Non-synonymous SNPs Functional Predictions (dbNSFP) (Liu et al., 2011). These methods are based on either protein sequence conservation (SIFT and Polyphen2) or DNA sequence conservation models (MutationTaster, PhyloP, and LRT) for non-synonymous variants. These scores range from 0 to 1, with 0 indicating variants being tolerant and 1 indicating highly deleterious. The information regarding clinical-association is extracted from ClinVar (Landrum et al., 2014), COSMIC (Bamford et al., 2004), DECIPHER (Firth et al., 2009), and OMIM (Hamosh et al., 2002) databases. The extracted information is in the form of clinical significance (clinSign) and phenotype from ClinVar, COSMIC ID, OMIM ID, and DECIPHER score (predicted probability and percentage) for the gene spanning the variant. A gene is categorized as haploinsufficient if DECIPHER_percentage score lies between 0 and 10%. Fourth level of information provided is variant/gene-drug association extracted from PharmGKB (Gong et al., 2008). In SeqVItA, the user may choose from two levels of drug assignment information for the variants: one is based on mapping with dbSNP ID (Stringent) and another based on mapping with the gene (flexible). The information provided include the name of the associated drug, type, and confidence level of action for a given variant/gene. These three categories of annotations has been used to obtain disease-associated variant prioritization from sequence data. A snapshot of the annotated file generated by SeqVItA is shown in Supplementary Figure S4.

In the analysis of multiple samples, if the user is interested in identifying genes carrying mutations across the samples (the variants may or may not be the same across samples), the function ***findRecurrentGenes*** is used to facilitate identification of variant-gene correspondence which can be outputted in a singular tabular format file, as shown in Supplementary Figure S5.

#### Prioritization

All the sequence variants detected in a patient's sample may not contribute to the disease and it would be helpful in identifying the important set of variants for therapeutics. In SeqVItA, the prioritization of sequence variants is based on the three categories of annotations discussed above. A variant is assigned “High” priority if the functional impact of the variant score is high (>0.65) from any one of the five resources (SIFT, Polyphen2, MutationTaster, LRT, and PhyloP), clinical association identified in at least one of the three resources (ClinVar, COSMIC, and OMIM), and variant-drug association identified in PharmGKB. A variant is assigned “Medium” priority if annotation is reported from any of the three categories. Any variant not meeting the above criteria is assigned “low” priority.

## Results and discussion

We discuss below the performance of SeqVItA on simulated data and real (cancer) data from 24 samples. In the simulated experiment, the effect of various parameters, *viz*., sequencing coverage, read length, minimum coverage threshold, base quality, type of SNVs and INDELs (homozygous or heterozygous), and size of INDELs (1–10) on the prediction accuracy is assessed. The performance of SeqVItA on real data is carried out by detecting somatic variants (both SNVs and INDELs) in paired exome sequence data (tumor and matched normal) from 24 liver cancer patients (SRP123031). Finally, we discuss ability of the “***annotate***” module in SeqVItA in analyzing the mutational landscape across 24 liver cancer patients, and prioritize the somatic variants to understand the probable role of novel variants in tumorigenesis.

### Analysis of simulated data

Genomic DNA sequence of size 16,569 bp (hg18 assembly) is considered for simulating the reads and is called “reference” sequence. In the simulated experiment, detection of both homozygous and heterozygous SNVs and INDELs is considered. To simulate homozygous condition we insert variations at random locations in a copy of the reference sequence and concatenate this “mutated reference” sequence to itself (to obtain the diploid genome), while the “mutated reference” sequence is concatenated to the “reference” sequence to simulate heterozygous condition. That is, four independent diploid genome sequences are constructed containing (i) 12 homozygous SNVs, (ii) 12 heterozygous SNVs, (iii) 4 different sizes (e.g., 1, 2, 5, and 10) of homozygous insertions and deletions, and (iv) 4 different sizes of heterozygous insertions and deletions. Reads are simulated (for Illumina sequencing in this case) using ART simulator (Huang et al., 2012) at three different sequencing coverages (20 ×, 40 ×, and 60 ×) for two different read lengths (50 and 100 bp), that is, a total of 6 experiments are conducted for the four diploid genomes constructed. ART is the most widely used simulator to generate synthetic NGS reads by emulating the sequencing process with built-in, technology-specific read error models and base quality value profiles parameterized empirically in large sequencing datasets. The reads are aligned to the “reference” sequence using Bowtie2 (Langmead and Salzberg, 2012). For each sequencing depth and read length, this exercise is repeated 50 times and the predictions are averaged over these repetitions. Variants detected using SeqVItA are then compared with three popular resources, BCFtools (Li, 2011), VarScan2 (Koboldt et al., 2012) and GATK HaplotypeCaller (McKenna et al., 2010).

In the simulated experiments, performance of SeqVItA is evaluated on two important parameters, *viz*., sequencing coverage and base quality. Base quality is given by Phred score of the base and only those reads that have a Phred score above a certain minimum quality score, Qbase is considered. We considered two base quality thresholds, 15 and 30 for the analysis. Minimum number of reads (with base quality > Qbase) required to make a call at a location is called minimum coverage threshold, RD_th (= 10, default). Another important parameter is the variant allele frequency (VAF), defined as the ratio of the number of reads supporting the variant allele to the total depth at that location. For the analysis, the threshold for variant allele frequency, VAF_th considered is 0.2. It is a useful parameter for filtering false positives resulting from sequencing errors.

The prediction accuracy of SeqVItA is evaluated by computing the Sensitivity (recall), Precision and F-score:

(5) $$\begin{array}{rcl} \text{Sensitivity} & = & \;\frac{TP}{TP\;+\;FN} \end{array}$$

(6) $$\begin{array}{rcl} \text{Precision} & = & \;\frac{TP}{TP\;+\;FP} \end{array}$$

(7) $$\begin{array}{rcl} \text{F}-\text{score} & = & \;\frac{2\;\times \;Sensitivity\;\times \;Precision}{Sensitivity\;+\;Precision} \end{array}$$

where True Positive (TP) is defined as the number of sequence variants correctly predicted, False Positive (FP) is the number of incorrect variants calls made, and False Negative (FN) is the number of variants missed, compared to the annotated list (Supplementary Tables S1, S2).

#### Performance evaluation of SeqVitA

##### Detection of SNVs and small INDELs

**Figure 2:**
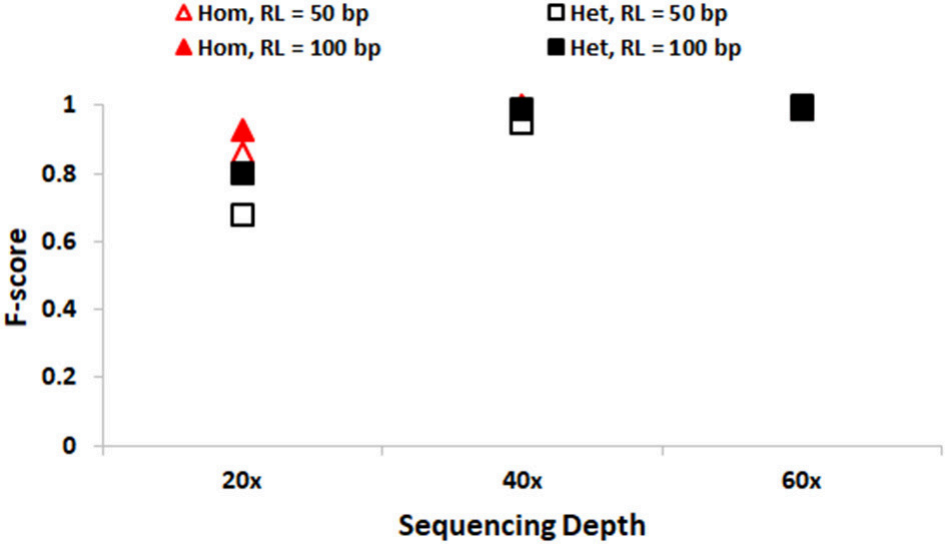
Performance of SeqVItA in detecting SNVs in simulated data shown. F-score values for detecting homozygous “triangle” and heterozygous “square” SNVs with read length 50 bp (empty symbols) and 100 bp (filled symbols). Minimum coverage threshold = 10 and Base quality ≥15.

We first discuss the performance of SeqVItA in detecting SNVs. In Figure 2, the prediction accuracies in detecting homozygous (“triangle”) and heterozygous (“square”) SNVs is depicted for two different read lengths, 50 bp (“empty symbols”) and 100 bp (“filled symbols”) and base quality score (≥15). As expected, the prediction accuracies are better with longer reads (100 bp) compared to smaller read lengths (50 bp), especially at lower sequencing coverage < 40×. This may probably be due to reliable alignment in the case of longer reads and suggests the need for higher sequencing coverage when the read lengths are small. It is easier to detect homozygous SNVs at low sequencing depth (20×), compared to the detection of heterozygous SNVs, irrespective of read length. It is observed that precision ~1 in the detection of both homozygous and heterozygous SNVs even at low sequencing coverage (20×) and small read lengths (50 bp), indicating the reliability of results. However, the recall values are seen to approach 1 only for high coverage data (≥ 40×) (see Supplementary Tables S3, S4).

**Figure 3:**
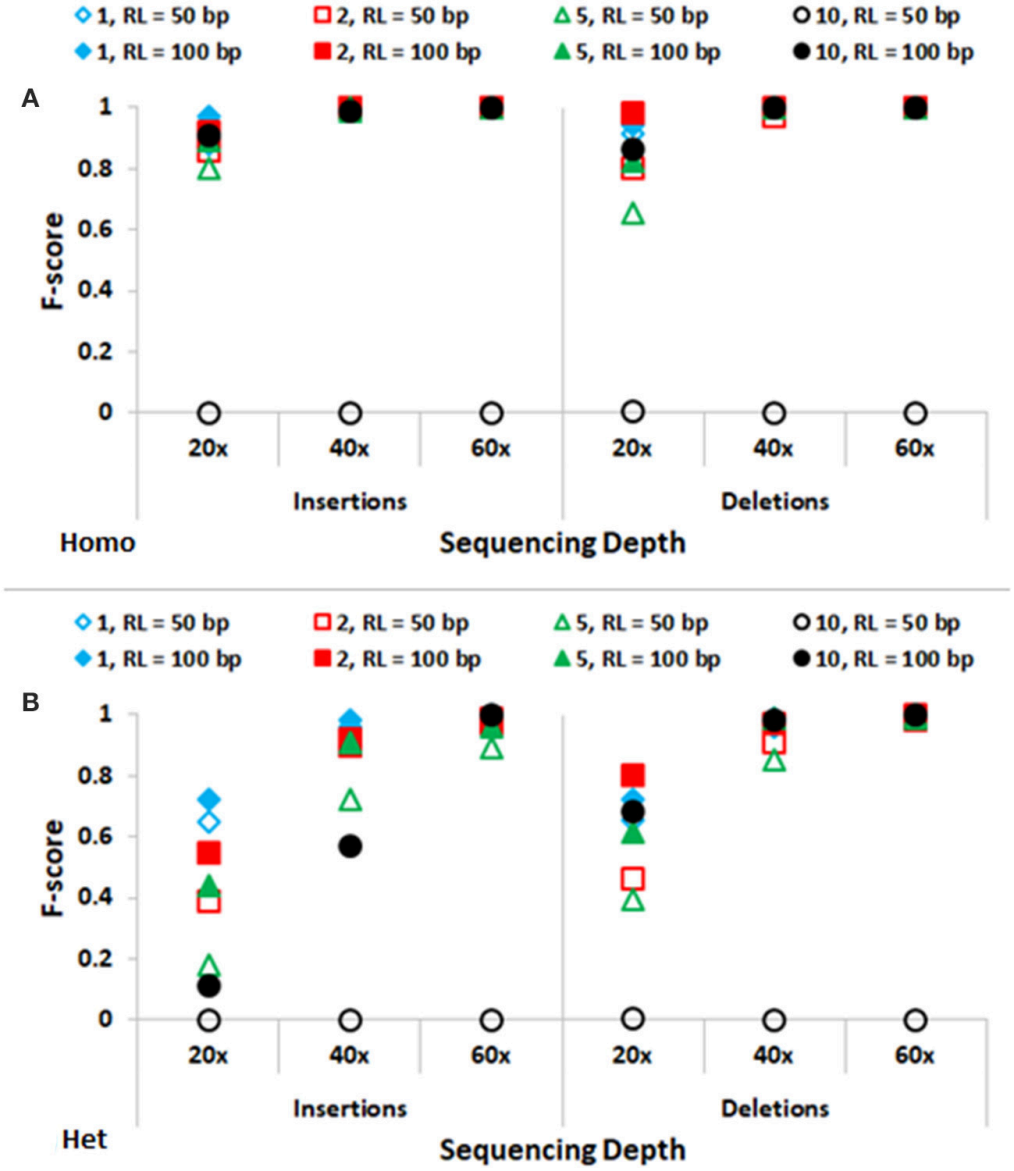
Performance of SeqVItA in detecting INDELs in simulated data shown. F-score values for predicting **(A)** homozygous (Homo) and **(B)** heterozygous (Het) INDELs of various sizes: 1 bp (“diamond”), 2 bp (“square”), 5 bp (“triangle”) and 10 bp (“circle”) for two read lengths 50 bp (empty symbols) and 100 bp (filled symbols). Minimum coverage threshold = 10 and Base quality ≥ 15.

In Figure 3, performance of SeqVItA in detecting homozygous and heterozygous INDELs of varying sizes (1, 2, 5, and 10 bp) is depicted, for two read lengths (50 and 100 bp), three sequencing depths (20 × , 40 × , and 60 ×) and base quality score (≥15). The performance of SeqVItA in detecting homozygous INDELs is summarized in Figure 3A. In this case also the prediction accuracy is observed to be good for high coverage data (≥40 ×) irrespective of the reads length. Poor performance at lower sequencing depth (20 ×) is likely to be due to the minimum coverage threshold of 10 reads not being met due to various biases in NGS data, *viz*., mappability bias (in low complexity regions) and GC bias. A clear dependence of the detection of longer INDELs on the read length is observed. It may be noted in Figure 3 that INDELs of 10 bp are missed with smaller read length (~50 bp), but are detected by increasing the read length to 100 bp. On further increasing the read length (200 bp) we were able to detect INDELs of larger sizes as well, *viz*., 15, 20, and 25 bp (results not shown). This is because the number of reads aligning in the vicinity of a large INDEL (≥10 bp) is considerably reduced and longer read lengths can tolerate larger gaps in the alignment, thus improving the performance. The performance of detecting heterozygous INDELs in Figure 3B is observed to be poor compared to that of homozygous INDELs, especially at 20 × sequencing coverage. We also observe dependence of prediction accuracy on the base quality threshold. Contrary to the expectation, on increasing the base quality cutoff value from 15 to 30, a dip in the performance is observed, especially at lower sequencing coverage (Supplementary Tables S5–S8). This is because a higher threshold for base quality may result in fewer reads supporting variant. These results suggest considering a higher base quality score if the sequencing coverage is ≥ 40 × .

##### Minimum coverage threshold

Accurate detection of SNVs and small INDELs is affected by various parameters and briefly discussed below. The distribution of reads is not uniform along the genome due to various reasons, *viz*., PCR artifacts leading to GC content bias, mappability bias, base quality, sequencing error, etc. As a consequence, read depth is not uniform across all the positions in the genome. Hence one needs to appropriately set the minimum coverage threshold, too high values may miss out true positive calls while too low values may increase the number of false positives. For data samples with sequencing depth ≥ 40× , a default minimum coverage threshold of 10 is observed to result in high accuracy (Figures 2, 3), both in the detection of SNVs and INDELs, irrespective of the size of reads or INDELs. However, the performance is relatively poor in the case of samples with sequencing depth 20 × . In this case, on decreasing the minimum coverage threshold to 5 (1/4th of average sequencing depth), the performance is seen to improve, especially for homozygous SNVs and short deletions (results not shown).

##### Read length

It may be noted from Figures 2, 3 that the performance is improved on using longer reads (100 bp) compared to 50 bp reads for sequencing depth <60× , especially in the detection of heterozygous SNVs and longer INDELs (≥5 bp). Large heterozygous indels (≥10 bp) are not detected even at 60 × sequencing depth with 50 bp read lengths.

##### Base quality

Base quality score is defined by the Phred score is inversely proportional to the probability of error in calling a base. Intuitively we expect higher base score to result in reliable predictions. However, we observe lower recall values on increasing the base quality score (Supplementary Tables S3–S8). This is because the number of reads available at a location are reduced, and if the sequencing coverage is low, this may not pass the minimum coverage threshold criteria required for variant calling.

Because of this inter-dependency between base quality score and minimum thresholds coverages one needs to set these parameters judiciously based on the available sequencing coverage of the data.

#### Comparison of SeqVitA with other tools

**Figure 4:**
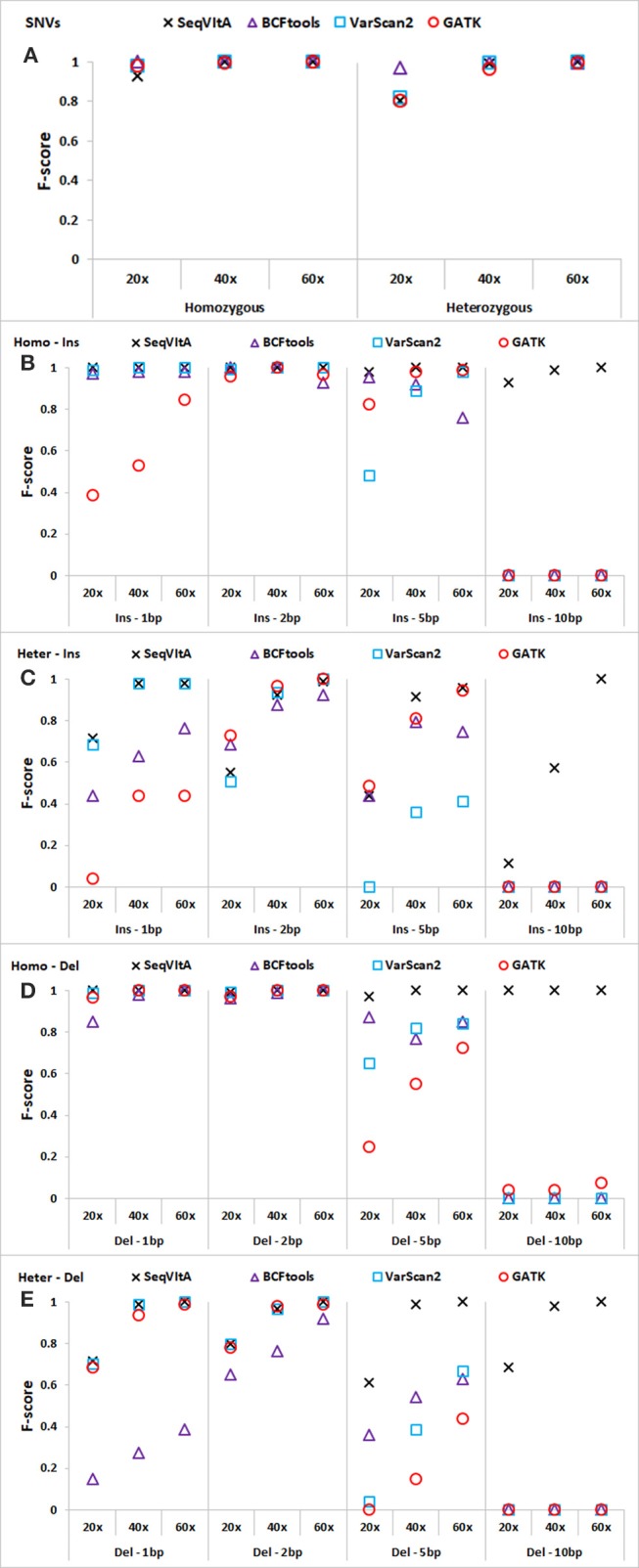
Performance comparison of SeqVItA with BCFtools, VarScan2 and GATK on simulated data at three sequencing depths 20 ×, 40 ×, and 60 × in detecting homozygous (Homo) and heterozygous (Het) **(A)** SNVs, **(B,C)** insertions (Ins), and **(D–E)** deletions (Del). Read length = 100 bp, and base quality threshold ≥ 15.

The performance of SeqVItA is compared with three popular sequence variant detection tools, *viz*., BCFtools (Li, 2011), VarScan2 (Koboldt et al., 2012), and GATK HaplotypeCaller (McKenna et al., 2010) on simulated data. The default parameters used in each of these four tools is summarized in Table 5. Performance is evaluated in the detection of both homozygous and heterozygous SNVs and INDELs (size ≤ 10 bp), at three sequencing depths (20 × , 40 × , and 60 ×). Read length is 100 bp in simulated experiments and base quality score is set to 15. Results are summarized in Figure 4 and in Supplementary Tables S9–S14.

**Table 5:** Various parameters considered for performance evaluation of SeqVItA on simulated data with three other tools.

	**SeqVItA**	**BCFtools**	**VarScan2**	**GATK**
Mapping quality bias correction	Recalibration of mapping quality: $Mq^{\prime }=\sqrt{\frac{Int-Mq}{Int}}\times Int$	Mann–Whitney *U*-test	–	Wilcoxon rank sum test
Mapping quality cut-off	20	20	20	–
Minimum coverage cut-off	10	–	8	–
Base quality cut-off	15	15	15	15
Germline *p*-value cut-off	0.01	–	0.01	–
Strand bias	Discard sites with < 10% or > 90% strandedness	Mann–Whitney *U*-test	Discard sites with < 10% or > 90% strandedness	Fisher exact test

In Figures 4A–E, we observe that the overall performance of SeqVItA is comparable to the other state-of-the-art tools in the detection of homozygous SNVs. BCFtool performs best in detecting heterozygous SNVs (at low coverage, 20×), while the performance of SeqVItA is better in detection of INDELs. It may be noted in Figures 4B–E that all the three tools failed to detect large INDELs (homozygous and heterozygous), even at 60 × coverage. The reason SeqVItA is able to detect larger INDELs is due to the recalibration of the mapping quality (equation 1) that handle mappability bias in the pre-processing step. Without pre-processing step, SeqVItA also fails to identify large INDELs. In general, performance of all the tools is better in detecting homozygous INDELs compared to heterozygous INDELs.

#### Computation time

SeqVItA is computationally very efficient compared to VarScan2 and GATK due to parallel implementation of the variant calling step using OpenMP. For a WES alignment file of size 14 GB (coverage: ~100×), SeqVItA took ~40 min to detect both SNVs and INDELs and is about 3 × faster than GATK (~2 hrs), ~2× faster than VarScan2 (~ 1 h 15 min) and is comparable to the time taken by BCFtools (~35 min). For comparing the computational efficiency, all the three tools are installed on a 64-bit Ubuntu machine with an Intel i7-4700MQ @2.4GHz processor. The minimum computational requirement for running SeqVItA is a desktop with 4 GB RAM running at 1.6 GHz and 500 GB disk space.

### Cancer data

A total of 26 Liver cancer patient samples and their matched control samples (average age: 52 years, range: 27–86 years) are downloaded from Sequence Read Archive database (Project ID: SRP123031). It is paired-end data of read length 76 bp obtained from targeted sequencing of 372 gene panel for Hepatocellular carcinoma (HCC), a primary type (70% cases) of liver cancer. The pre-processing of data is carried out using NGC QC Toolkit and reads with quality scores < 30 are removed. Due to an error in the pre-processing step, two samples (patients 3 and 25) are discarded, leaving 24 HCC patient samples for subsequent analysis. The pre-processed reads are then aligned with the human reference genome (hg19 assembly) using Bowtie2. Post-alignment processing is carried out using Picard tools (http://broadinstitute.github.io/picard/) to identify and mark PCR duplicates (which are then ignored in the variant calling step). For each processed file, sequence variants are identified using “***somatic***” module of the pipeline and analyzed using the “***annotate***” module. Reads with mapping quality, Mqread < 20 are discarded, mapping quality correction was applied (using Mqcorr = 50 in eqn 1), and sites with minimum coverage, RD_th (≥50) in both tumor and normal samples are considered for variant calls.

### Mutation landscape of sequent variants in liver cancer patients

The variants detected by the Somatic module are labeled germline, somatic, LOH and “Unknown” for the 24 paired tumor-control samples. As expected, majority of the predicted SNVs in the 24 patients are germline variants (~90%), followed by LOH (~4.9%) and somatic (~4.1%) variants, with an average of 2007 high confident SNVs per tumor sample. Small INDELs of size ranging from 2 to 27 bp are also identified in these tumor samples with the number of insertions (97 per sample, 50.5%) and deletions (95 per sample, 49.5%) nearly equal. On average, 182 high confident small INDELs are predicted per sample and of these, like SNVs, majority of the INDELs are of germline origin (~85.5%), followed by LOH (~8%) and somatic (~5.5%) (Supplementary Table S15).

### Analysis of somatic sequence variants

Since somatic sequence variants are most likely to be responsible for tumorigenesis, these are filtered for further functional analysis using the “***splitVCF***” function and their distribution in the genic regions is identified (Supplementary Figure S6). It is observed that a large fraction of the variants lie in the intronic regions (59%) followed by promoter (18%), 3′ UTR (6%) and 5′ UTR (2%) regions. Among the variants in the coding regions, most common are missense variants (10%), followed by synonymous (4%), nonsense (0.7%), frameshift (0.5%), and splice site (0.2%) variants.

**Figure 5:**
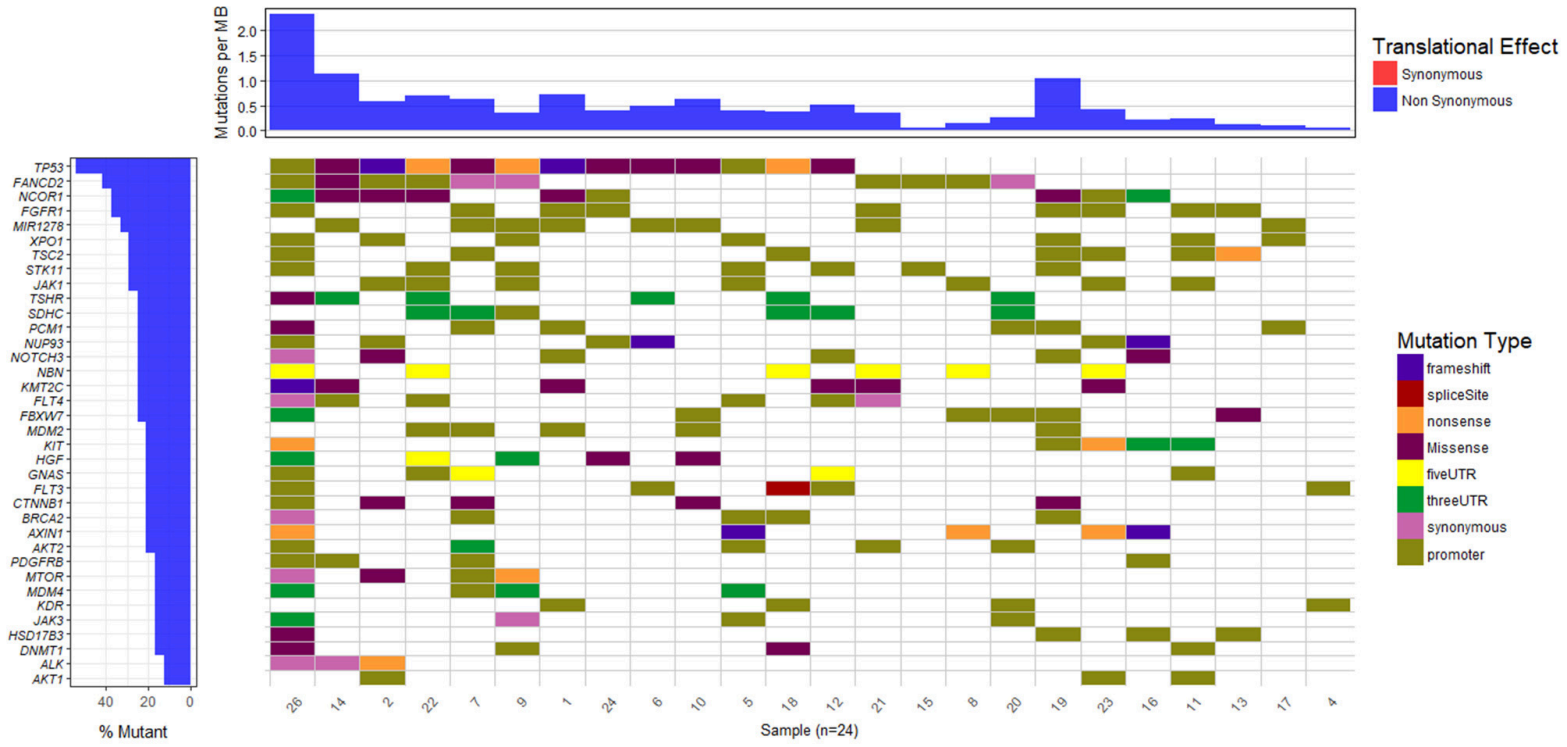
Mutational landscape of somatic sequence variants identified in 24 HCC patient samples (intronic and intergenic variants excluded). Each column corresponds to each patient sample and each row represents a gene.

The waterfall plot in Figure 5 exhibits the mutational landscape of genes associated with recurrent sequence variants (identified in at least 15% of the patients). It is observed that the tumor suppressor gene TP53 carries mutations in 13 out of 24 samples (54%) though the location and/or type of mutation vary from patient-to-patient. For example, patient number 9, 18, and 22 exhibit nonsense mutation C>A, at locations chr17:7577046, chr17:7579521, and chr17:7578188, respectively; patient number 1 and 2 exhibit frameshift mutations (G[AA/-], chr17:7578212 and C[A/-], chr17:7578231, respectively), while patients 5 and 26 have mutations in the promoter regions of TP53 gene (A>C, chr17:7577407, G>A, chr17:7577427 in patient 5 and G>T, chr17:7579619, A>G, chr17:7579825, in patient 26). In the remaining patients, the variants are observed in the intronic regions of TP53 gene.

We also observe other well-known HCC associated genes, e.g., NCOR1 (38%), AXIN1 (21%), MDM2 (21%), CTNNB1 (21%), MTOR (17%), mutated in a significant number of patients. Gene NCOR1 is known to exhibit strong tumor suppressor activity, preventing tumor cell invasion, growth, and metastasis. Genes TP53 and MDM2 participate in G2/M DNA damage checkpoint of the cell cycle, while genes AXIN1 and CTNNB1 are involved in Wnt/β-catenin signaling pathway, and MTOR mediates cell proliferation and differentiation through MAPK signaling pathway. The potential role played by these pathways in cancer is well-studied and aberrations in these candidate genes are known to initiate liver tumorigenesis (Guichard et al., 2012; Meng et al., 2014, p. 2; Satoh et al., 2000; Ou-Yang et al., 2018, p. 1). Out of nine genes reported to be involved in HCC in the peer-reviewed database, Atlas of Genetics and Cytogenetics in Oncology and Hematology (Huret et al., 2013), seven have been identified in few of these 24 HCC patients. These include tumor suppressors TP53 (54%), AXIN1 (21%), SMAD2 (13%), CDKN2A (13%), RB1 (8%), and oncogenes CTNNB1 (21%) and CCND1 (8%).

### Principal component analysis based on mutated genes

To explore if the 24 HCC patients share the same set of mutated genes or they exhibit different mutational profile, we carried out principal component analysis on these genes. The objective is to identify groups of patients sharing common variants and disease-causing pathways.

**Figure 6:**
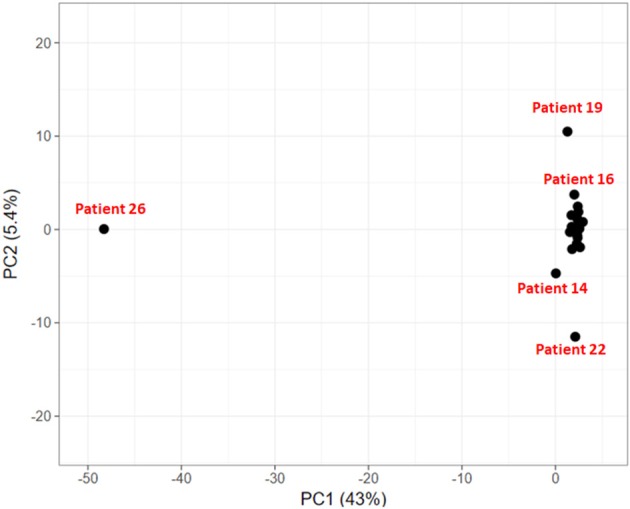
Clustering of HCC patients based on somatically mutated genes.

In the PCA plot in Figure 6, we observe that majority of patients form a single group, while patient 19, 14, and 22 show deviation from this group and patient 26 is a clear outlier. Analysis of Patient 26 data resulted in a completely different profile with a much larger set of variants compared to the rest of the 23 patients. It had 787 sequence variants across 156 genes, while the average number of variations observed in other patients ranges from 29 to 126 variations. Accumulation of mutations is a known somatic mutational signature in liver cancer (Letouzé et al., 2017) and noting the age of patient 26 (86 yrs), it is not surprising. Analysis of the remaining 23 patient samples clearly also revealed the dependence of number of somatic mutations as a function of age. On average, patients with age ≥ 55 years exhibited ~160 somatic mutations, while patients < 55 years showed ~57 mutations per patient. Patient 19 and patient 22 also showed relatively higher number of mutations, 124 and 126, respectively (Patient 19: 56 yrs and Patient 22: 60 yrs). However, the set of mutated genes is very different in the two cases. Patient 14 exhibited slightly larger number of variants (74) and had a good overlap with that of Patient 22. This analysis indicates that though all the patients are diagnosed with HCC, they exhibit different genetic profiles, suggesting that same drug/dosage may not be suitable to all the 24 patients. Thus, there clearly exists a need for personalized screening of the genetic variants and associated functional impact for accurate treatment. Based on PCA, the 24 patients are clustered in four groups (G1: Patient 26, G2: Patient 19, G3: Patients 22 and 14, and G4: remaining 20 patients). We first discuss below the analysis of the larger group of 20 patients to understand the role of common mutations in HCC.

### Recurrent somatic variants and their role in liver cancer

To identify common somatic variants and associated genes in group G4 patients, genes carrying mutations in at least 25% of the patient samples are considered. These common set of mutated genes, summarized in Table 6, are probable diagnostic and prognostic markers for HCC patients of this group (complete listing of sequence variant hotspots is given in Supplementary Table S16). These span exons, introns, promoters, or 5′ and 3′ untranslated regions, and may directly or indirectly contribute to liver tumorigenesis. It is observed that not all patients carry the same mutation in a gene. For e.g., the characteristic TP53 mutation in 7th exon (C>A, chr17:7577534, R249S) is observed only in two patients, 7 and 12 (i.e., 20% prevalence). Deletions, leading to frameshift (patients 1 and 2), and diverse substitutions in coding regions are observed in remaining patients, while patient 5 is observed to carry two intronic variations. The direct role played by these TP53 variants in HCC is still unknown. Similarly, location and type of variations in FGFR1, FANCD2, and MIR1278 (35%), JAK1 (30%), and NCOR1, NUP93, XPO1, SDHC, and TSC2 (25%) genes are observed to vary from patient-to-patient (see Supplementary Table S16).

**Table 6:** Summary of recurrent genes exhibiting somatic mutations in at least 25% of liver cancer patients.

**Gene**	**No. of patients with SVs**	**Patient IDs**	**Total No. of SV hotspots**	**Total SNVs**	**Total INDELs**
TP53	10	1,2,5,6,7,9,10,12,18,24	10	8	2
FGFR1	7	1,7,11,13,21,23,24	5	5	0
FANCD2	7	2,7,8,9,15,20,21	5	4	1
MIR1278	7	1,6,7,9,10,17,21	7	7	0
JAK1	6	2,5,8,9,11,23	5	5	0
NCOR1	5	1,2,16,23,24	9	9	0
NUP93	5	2,6,16,23,24	4	2	2
XPO1	5	2,5,9,11,17	3	2	1
SDHC	5	7,9,12,18,20	3	3	0
TSC2	5	7,11,13,18,23	4	4	0

**Figure 7:**
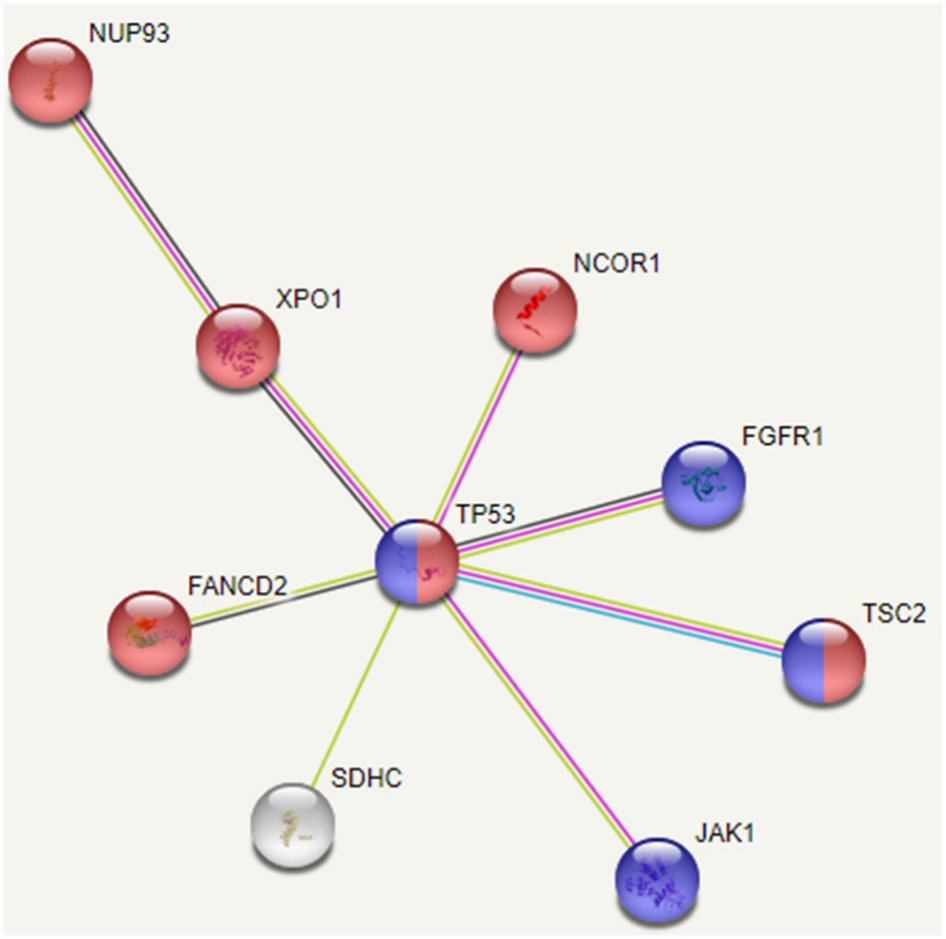
Interaction between recurrently mutated genes from STRING database. Pathway enrichment analysis of these mutated genes indicate that cell cycle (shown in red) and PI3K/AKT (shown in blue) pathways are affected.

Based on the interactions extracted from STRING database (Szklarczyk et al., 2017) between these mutated genes and pathway enrichment, we observe that cell cycle (FDR: 0.007) and PI3K-AKT (FDR: 0.002) pathways are overrepresented in this group, as shown in Figure 7. Genes TP53, FGFR1, JAK1, and TSC2 participate in PI3K/AKT pathway, an important pathway involved in cell growth and proliferation, and activated in many tumor types including HCC. It may be noted that the tumor suppressor TP53 affects both the pathways. Dysregulation of cell cycle plays a key role in promoting liver carcinogenesis through evasion of growth suppressors (TP53 and NCOR1), sustaining proliferative signaling (FGFR1, XPO1, and TSC2), chromosomal instability (FANCD2 and NUP93), invasiveness, survival and metastasis (Bisteau et al., 2014). Out of 20 patients, 14 show diverse mutations (coding or intronic) in genes that directly participate in PI3K/AKT pathway.

Thus, we see that though the 20 patients of group G4 carry different SVs, these affect mainly cell cycle and PI3K/AKT pathways. Our analysis also revealed some novel variants, not yet associated with HCC; G>A, chr1:193104827 (5′ UTR) in MIR1278 (7 patients), G[GAAAATC/-], chr2:61713209 (intron) in XPO1 (5 patients); C>G, chr1:161332346 (3′ UTR) in SDHC (4 patients), C>T, chr8:38282294 (intron), and C>G, chr8:38272542 (intron) in FGFR1 (4 patients each). These may be probable prognostic markers and their role in HCC may be further investigated on larger sample data.

### Comparative analysis of patient-specific sequence variants

We next show functional analysis of sequence variants identified using SeqVItA in patient 9 (a representative of large cluster) and patients 19 and 22 (exhibiting variants differing from that of large cluster) i.e., a comparative analysis of the member of the three groups G2, G3, and G4. In Table 7, the variant genes and their functional impact on pathways and drug response are summarized for patients 9, 19, and 22. The first striking difference observed is the number of genes carrying variants. It is much lower in patient 9 (39 yrs) compared to patients 19 (56 yrs) and 22 (60 yrs), probably because of the difference in their age. Further, they share very few common mutants. Below we discuss the analysis of “High” and “Medium” priority genes to understand pathways and processes affected that may lead to tumorigenesis in the three groups (complete annotations in Supplementary Table 2.XLSX).

**Table 7:** Summary of sequence variants and their functional role identified in HCC patient 9 (39 yr old, male), patient 19 (56 yr old, male), and patient 22 (60 yr old, male).

**Patient Number**	**9**	**19**	**22**
Number of mutated genes	44	77	69
High/Medium priority genes	TP53, FANCD2, SDHC, MTOR, TPMT, DNMT1, GSTA1, CYP2B6 (8)	CTNNB1, MSH1, FBXO11, MSH6, MAP3K1, KMT2C, BRCA2, NCOR1, BCR, ARID2, CARD11, ERG, GRM3 (13)	SDHC, ATR, FCGR2B, ESR1, HGF, CDKN2A, ATM, TSHR, TP53, NCOR1, NF1, KEAP1, BCR, MSH2 (14)
Known oncogenes	MTOR	CTNNB1, BCR, CARD11, ERG	CDKN2A, BCR
Known tumor suppressors	TP53, DNMT1	NCOR1, BRCA2, KMT2C, ARID2	SDHC, ATR, ESR1, ATM, TP53, NCOR1, NF1, KEAP1
Key pathways affected	TP53: cell cycle MTOR: Upregulation frequently observed in HCC, MTOR –| PTEN, IGF and EGF pathways DNMT1: Methylates PTEN promoter –| PTEN activation of PI3K/AKT/mTOR pathway	CTNNB1: Wnt pathway → proliferation and survival FBXO11: Tumor initiation & progression MSH1 & MSH6: DNA mismatch repair MAP3K1: JNK & Erk pathway → proliferation KMT2C, ARID2: Chromatin modifications MAP3K1, MSH6, BRCA2, NCOR1, CTNNB1, BCR: Cell cycle GRM3: GPCR signaling pathway → proliferation	KEAP1: Oxidative stress → proliferation and survival in HCC HGF: Overexpressed HGF binds with c-Met proliferation & survival in HCC through c-MET signaling pathway CDKN2A: G1/S cell cycle ESR1: Estrogen signaling pathway → proliferation TP53, CDKN2A, ATR, ATM: p53 signaling pathway, replicative senescence, cell cycle
Variant-Drug associations (PharmGKB)	Mutations in GSTA1 & CYP2B6 affect enzymatic activity of drugs → lower efficacy	CTNNB1(A>G): Ethnic-specific, wild-type (AA) associated with better response to CTD therapy, not significant in heterozygous condition	ESR1: Increased risk of azoospermia in childhood cancer survivors when treated with alkylating agents and cisplatin

### Patient-specific sequence variants

We observe different set of oncogenes and tumor suppressor genes mutated in the three patients. Different signatures of somatic mutations indicate probable differences in the processes underlying the initiation, growth and survival of tumorigenesis in these patients.

From Table 7, we observe that in patient 9, mutations in oncogene mTOR (nonsense mutation) and tumor suppressors TP53 (nonsense mutation) and DNMT1 (2 bp intronic deletion) are likely candidates for tumorigenesis. Genes TP53 and mTOR participate in PI3K/AKT pathway involved in cell growth and proliferation, and are known to be activated in many tumor types including HCC. Mechanistic target of rapamycin (MTOR) is a highly conserved serine/threonine protein kinase that plays a crucial role in cell proliferation, differentiation, metabolism, and aging via two structurally and functionally distinct multi-protein complexes: mTORC1 and mTORC2. In earlier studies mTOR pathway has been shown to be upregulated in ~50% liver cancer patients and associated with deregulation of PTEN, IGF, and EGF pathways, key players in HCC (Matter et al., 2014). Tumor suppressor TP53 participates in G2/M DNA damage checkpoint and its activity is arrested due to a nonsense mutation in patient 9. This affects the p53 signaling pathway which responds to DNA damage by arresting the cell cycle and facilitates DNA repair by transactivating DNA repair genes. DNA methylation is known to play an important role in epigenetic gene regulation and aberrant DNA methylation patterns are commonly found in tumors. The other tumor suppressor gene, DNA methyltransferase 1 (DNMT1) is shown to hypermethylate promoter region of tumor suppressor, PTEN, resulting in decreased activity of the gene in a study on rat model (Bian et al., 2012). Decreased activity of PTEN leads to triggering of PI3K/AKT/mTOR pathway and uncontrolled growth and proliferation of tumor cells in the liver (Golob-Schwarzl et al., 2017). The 2 bp deletion observed in the intronic region of DNMT1 gene in patient 9, is reported in COSMIC to be participating in adenocarcinoma, though indicated to be benign in ClinVar. Our results suggest that the effect of this variant in HCC may be further investigated. Thus, we observe that the major pathway affected in patient 9 is PI3K/AKT/mTOR signaling pathway.

The mutational profile of Patient 19 includes four oncogenes CTNNB1 (missense/intronic), BCR (missense/synonymous/intronic), CARD11 (missense), ERG (missense) and four tumor suppressor genes NCOR1 (2 missense), BRCA2 (intronic), KMT2C (intronic), ARID2 (nonsense). The missense mutation in CTNNB1 observed in Patient 19 is well-characterized in several cancers, including HCC and affects phosphorylation of β-catenin protein, preventing its degradation. Accumulation of excessive β-catenin in cells pushes the protein into the nucleus initiating uncontrolled growth and differentiation of tumor cells via Wnt signaling pathway. Oncogene BCR is involved in activating ERK pathway, a key kinase pathway that maintains cell cycle, and aberrations in the gene are associated with proliferation, differentiation, and inflammation. Protein encoded by CARD11 interacts with BCL10, acts as a positive regulator of cell apoptosis and activates the NF-κB pathway. The NF-κB protein complex acts as tumor promoter in inflammation-associated cancers, including HCC. Oncogene ERG and tumor suppressor gene NCOR1 are important transcriptional regulators. While ERG participates in embryonic development, cell proliferation, differentiation and apoptosis, NCOR1 interacts with nuclear receptors and other transcriptional factors. NCOR1 is shown to exhibit strong tumor suppressor activity, preventing tumor cell invasion, growth, and metastasis in mouse models (Fozzatti et al., 2013). The mRNA expression levels of NCOR1 are identified to be decreased in human liver cancer, either due to mutations or deletion of the gene (Martínez-Iglesias et al., 2016). Tumor suppressor BRCA2 is involved in DNA damage repair, while KMT2C and ARID2 (frequently mutated in HCC) participate in chromatin modifications. Gene KMT2C encodes for an enzyme histone methyltransferase that methylate lysine 4 of histone H3 and is involved in epigenetic transcriptional activation. Gene ARID2 is also involved in transcriptional activation and repression and is required for the stability of SWI/SNF chromatin remodeling complex SWI/SNF-B. Mutations in ARID2 have been directly associated with hepatocellular carcinogenesis. Recently, histone deacetylase inhibitors that affect the epigenetic pathway are used for therapeutics in HCC. Thus, our analysis of mutational profile reveals disruption of NF-κB, Wnt, and ERK signaling pathways, cell cycle, epigenetic and chromatin modifications, responsible for HCC initiation and progression in patient 19.

Patient 22 exhibits mutations in two oncogenes, CDKN2A and BCR, and eight tumor suppressor genes, *viz*., SDHC, ATR, ESR1, ATM, TP53, NCOR1, NF1, KEAP1. A detailed analysis of the variants in these genes reveals that c-MET signaling pathway is affected via a synonymous SNV in Hepatocyte growth factor (HGF), leading to initiation, proliferation, and survival of hepatic tumor cells. HGF on binding with c-MET results in a number of molecular events leading to the activation of MAPK (cell proliferation), PI3K/AKT/mTOR (cell survival) and Rac1-Cdc42 (cell mobility and cytoskeletal changes) signaling pathways (Goyal et al., 2013). A missense mutation in tumor suppressor, Kelch-like ECH associated protein 1 (KEAP1) is also identified in patient 22, which affects KEAP1-NRF2 pathway, a key regulator to cytoprotective responses, oxidative, and electrophilic stress. Mutations in KEAP1 are identified to disrupt the KEAP1-NRF2 regulatory system by increasing NRF2 levels and thereby affecting cancer cell proliferation and survival (Kansanen et al., 2013). In addition to oncogene CDKN2A (missense), tumor suppressors TP53 (nonsense), ATM (splice-site variant) and ATR (missense) participate in disruption of the cell cycle. While TP53 participates in G2/M DNA damage checkpoint, CDKN2A participates in G1/S DNA damage checkpoint of the cell cycle. Aberration in these genes leads to uncontrolled cell proliferation, increased survival, and genomic stability. Genes ATR and ATM are involved in DNA damage response through phosphorylation of cell cycle checkpoint kinases, CHK1, and CHK2 genes, respectively, in p53 signaling pathway. These mutations clearly indicate the disruption of cell cycle pathway in patient 22 and numerous signaling pathways, c-MET, MAPK, PI3K/AKT/mTOR, and p53.

The annotations for predicted variants using SeqVItA in patient 19 (114 somatic SNVs and 10 somatic INDELs) are compared with two annotation tools, ANNOVAR and CADD (which also prioritizes the variants). The output from variant calling step in SeqVItA was given as input to ANNOVAR (after modifying it in desired format). Since a number of resources integrated in ANNOVAR and SeqVItA are common, DNA conservation scores obtained from MutationTaster, LRT, and PhyloP, protein conservation scores from SIFT and PolyPhen2, disease-association relationships from ClinVar, OMIM, and COSMIC, and identification of known variants from dbSNP, identified by the two tools were the same. In Patient 19, 25 variants reported to span promoter regions in SeqVItA were not annotated in ANNOVAR, while allelic frequencies across various ethnic groups (1000 Genome Project and ExAc) were not provided by SeqVItA. All other gene-specific variants had same annotations from the two tools. Major difference between the annotations from SeqVItA and ANNOVAR is the variant-gene-drug associations from PharmGKB in SeqVItA. This is a very important feature and would assist the clinician in designing individulized therapy based on the effect of variant on the drug (efficacy, toxicity, and metabolism). Also, ANNOVAR does not prioritize the variants. For this we compared the prioritized variant list of patient 19 from SeqVItA with that provided by CADD. We observe that 9 out of 10 top-CADD scored variants were identified as High or Medium priority variants by SeqVItA. These results clearly indicate the reliability of SeqVItA with other state-of-the-art tools.

### Mutation drug-response associations

Thiopurine S-methyltransferase (TPMT) enzyme catalyzes S-methylation of aromatic and heterocyclic sulfhydryl compounds present in immunosuppressants (e.g., azathioprine) and antineoplastic drugs (e.g., 6-mercaptopurine and 6-thiogauanine). It is known to exhibit reduced enzymatic activity in patients with specific genetic variations in 5th, 7th, and 10th exons, and is an important pharmacogenetics biomarker (Asadov et al., 2017). Patient 9 carries a synonymous mutation (G>A, chr6:18139214) in 6th exon for which no effect on TPMT enzyme activity is reported, suggesting the suitability of these drugs in this case. Glutathione S-transferase A1 (GSTA1) is involved in detoxification of toxic molecules such as oxidative stress products, prostaglandins, chemical carcinogens, and therapeutic drugs, and mutation in this gene is associated with accumulation of toxic molecules with high risk of liver cancer (Akhdar et al., 2016). In PharmGKB four sets of drugs (or combination of drugs) associated with this SNV are reported, *viz*., (i) doxorubicin, (ii) cyclophosphamide, doxorubicin, prednisone, rituximab, vincristine, (iii) busulfan, and (iv) cisplatin and cyclophosphamide. For the first two categories, efficacy of the drug(s) is reported to be affected due to the variant in GSTA1 (Rossi et al., 2009). Additionally, elimination (metabolism) of drug busulfan, and toxicity or adverse drug response (as anemia) of cisplatin and cyclophosphamide are lower in the variant compared to wildtype GSTA1 (Khrunin et al., 2012; ten Brink et al., 2013). Of these, doxorubicin and cisplatin are commonly used therapeutics for HCC (Li et al., 2016), and may be given to patient 9 with appropriate dosage. Cytochrome P45 2B6 (CYP2B6) is a monooxygenase that catalyzes many reactions involved in drug metabolism and specifically known to metabolize a number of xenobiotics including antineoplastic drugs such as cyclophosphamide and ifosfamide, antidepressant drugs such as bupropion and sertraline. A missense SNV observed in exonic region of CYP2B6 gene (rs2279343) in patient 9 has a high log-ratio test (LRT) score (~0.99) indicating it is highly deleterious. In ClinVar and OMIM, the mutation is associated with efavirenz drug response and lower doses of the drug are advised in Japanese HIV patients with homozygous SNV to reduce drug toxicity (Tsuchiya et al., 2004). Thus, we show that a detailed analysis of the mutation profile of a patient can help in providing personalized therapy.

In patient 19, an intronic variant in CTNNB1 (rs4135385) is associated with drugs lenalidomide, cyclophosphamide, thalidomide, and dexamethasone (CTD) (efficacy, toxicity/ADR), with an inhibitory effect on tumor cell proliferation and inducing apoptosis in multiple myeloma (level 3 confidence). The distribution of the genotype frequency is population-specific, e.g., homozygous-reference (AA) is widely observed in healthy individuals of Poland and Saudi Arabia, while homozygous-variant (GG) is dominant in Chinese population. Efficacy of these drugs is high when the genotype is wild-type (AA) and no statistically significant response is observed for heterozygous condition (AG). However, patients with wild-type allele develop neutropenia as a side effect of the lenalidomide therapy (Butrym et al., 2015).

Also, intronic variant (rs2207396) in ESR1 observed in patient 22 is known to have an association with alkylating agents and cisplatin. In a study (Romerius et al., 2011), the effect of this variant in ESR1 analyzed in cancer survivors showed an increased risk of azoospermia in childhood cancer survivors when treated with alkylating agents and cisplatin, specifically with heterozygous (GA) genotype compared to homozygous (GG/AA) condition.

Though the mutations and corresponding drug responses in patient 9 (TPMT, GSTA1, and CPY2B6), patient 19 (CTNNB1) and patient 22 (ESR1) are not directly shown in HCC, these associations indicate that along with factors such as age, organ functions and tumor biology, genetic constitution of the patients may also affect the efficacy of the drugs used in treatment (20 to 95% variability). This clearly indicates the need for screening the patient genotype prior to devising the treatment regime.

### Comparison with VarScan2 and mutect2

To compare the performance of SeqVItA on real data, the aligned BAM files of patient 19 was considered as input to Mutect2 and the corresponding mpileup format file as input to SeqVItA and VarScan2.

**Figure 8:**
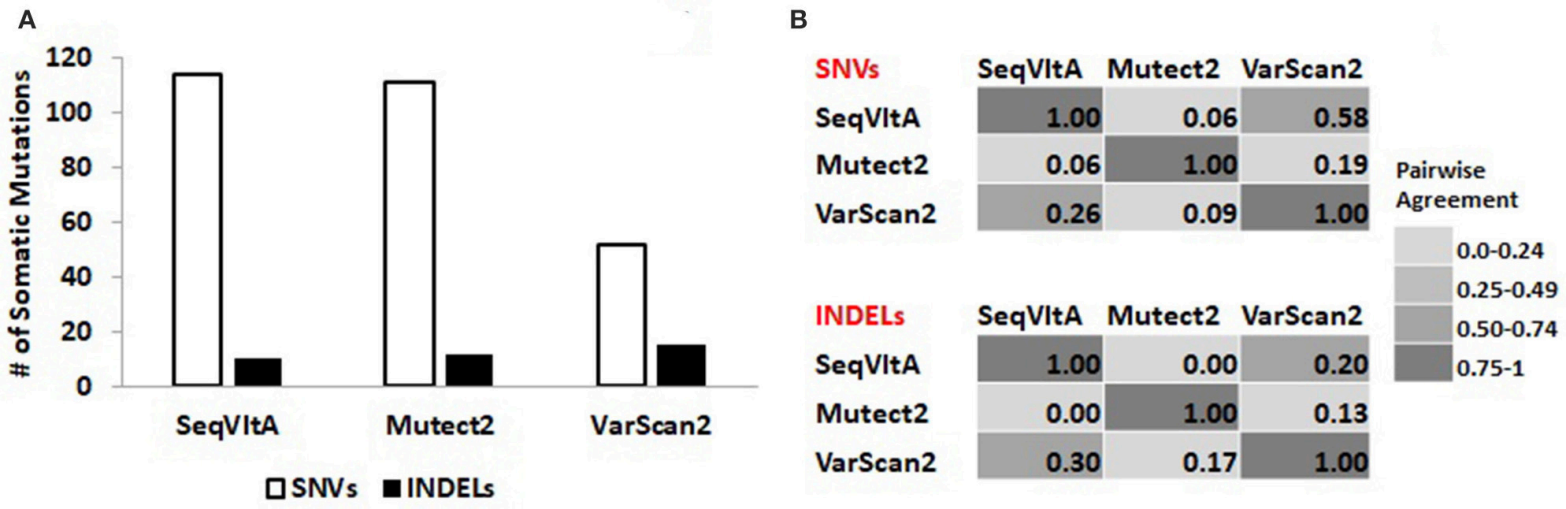
**(A)** Total number of somatic variants called and **(B)** Pair-wise agreement (0–1 scores) between SNVs and INDELs predicted by SeqVItA, Mutect2, and VarScan2.

From Figure 8A, we observe a clear variation in the total number of somatic variants predicted and their overlap between the three tools: VarScan2 (52, 15), SeqVItA (114, 10), and Mutect2 (111, 12), for SNVs and INDELs respectively. A similar observation was made by Krøigård et al. (2016) in performance evaluation of nine variant callers for the detection of SNVs and small INDELs. From Figure 8B we observe higher agreement between VarScan2 and SeqVItA results, probably because of the same heuristic approach with hard cut-off thresholds used in both the cases. The difference between their prediction outputs is probably because of strandedness bias considered in SeqVItA (but not in VarScan2). The mappability correction was not applied in Mutect2 which may probably be one of the reasons for its poor overlap with the other two tools, apart from the difference in the approach. The variants detected by a single tool are likely to contain many false positives and hence should be cross-validated either by predictions form other tools or based on annotations.

Variants predicted by the three tools spanning protein-coding genes are identified: SeqVItA (85 genes), Mutect2 (83 genes), and VarScan2 (51 genes). These genic-variants are further filtered based on the functional relevance of the variants/genes in liver cancer. We observe 10 genes commonly identified by the three tools. These include missense mutations in CTNNB1, CARD11, and GRM3 genes and nonsense mutation in ARID2 gene at the same loci, significance of which has been discussed in the previous section in Table 7. Additionally, SeqVItA and VarScan2 share common mutations in KMT2C (intron), NCOR1 (missense), and BCR (missense and synonymous) genes, but these are missed by Mutect2. VarScan2 did not predict any variant that has a known direct effect on the drug, while, Mutect2 identified a missense mutation (rs1801394) in gene MTRR that is associated with drug methotrexate. The heterozygous condition (identified in Patient 19) of this mutation is associated with higher levels of drug toxicity in childhood acute lymphoblastic leukemia and lymphoma (Huang et al., 2008). All the above discussed variants had scaled-score > 20 in the CADD annotation and prioritization tool, indicating their deleteriousness.

## Conclusion

Precision medicine is an emerging approach that considers individual genetic variability to explain differences in disease susceptibility, progression, and reaction to drugs at the population level. For disease prognosis, analysis of multiple genes quickly, and sensitively from small sample quantities is desirable, which is now possible with the advent of Next Generation Sequencing (NGS) techniques. However, to achieve this, there is a need to decode large genetic data more rapidly and accurately. Single nucleotide variants (SNVs) and small insertions and deletions (INDELs) are the most prevalent form of genetic variants and have been shown to play a potential role in the predisposition of disease and contribute to variable drug response in individual patients. In this study we show the efficacy of our tool SeqVItA in detection and functional analysis of SNVs and small INDELs in NGS data. Performance evaluations of SeqVItA on simulated data suggest high sequencing depth (≥40 ×) and larger read length (≥100 bp) for reliable prediction of variants. Compared to other popular methods for variant detection, SeqVItA is able to accurately detect larger INDELs (5 bp) because of mapping quality recalibration of reads in the pre-processing step. In the analysis of 24 liver cancer patients, importance of annotations extracted from various resources to prioritize the variants is discussed. These are confirmed by screening the literature for their role in liver cancer. Detailed comparative analysis of three patient samples is carried out to show the applicability of the proposed tool in personalized therapeutics. In the analysis of cancer data, one needs to take care of purity, ploidy, and heterogeneity of tumor samples which may affect the prediction accuracy of sequence variants. Further, presence of larger structural variants such as copy number variations, inversions and translocations should be taken into account as these may affect the detection of small sequence variants. One major limitation in SeqVItA's pipeline is non-availability of population-specific allele frequencies (e.g., 1000 Genome project) which can help in screening for rare variants. Currently in SeqVItA prioritization of predicted variants is based on a simple cumulative scoring scheme. There is scope for improving variant filtering using statistical or network-based approaches. SeqVItA is actively developing and we hope to expand the features of the annotation module to even include annotations for variants from non-genic regions.

## Author contributions

PD investigation, software, validation, writing original draft. SS methodology, software, validation. NP conceptualization, supervision, writing, review and editing.

### Conflict of interest statement

The authors declare that the research was conducted in the absence of any commercial or financial relationships that could be construed as a potential conflict of interest.
