# Supplementary material for: SeqVItA: Sequence Variant Identification and Annotation Platform for Next Generation Sequencing Data
Source: Front Genet. 2018 Nov 14;9:537. doi: 10.3389/fgene.2018.00537 (PMC6247818; doi:10.3389/fgene.2018.00537)
Supplement: Supplementary file 1 [file Table_1.DOCX]

Supplementary Material

**SeqVItA: Sequence Variant Identification and Annotation Platform for Next Generation Sequencing Data**

Prashanthi Dharanipragada, Sampreeth Reddy Seelam, and Nita Parekh*

*** Correspondence:** Dr. Nita Parekh: nita@iiit.ac.in

1. **Supplementary Tables**

**Supplementary Table S1: Location of Homozygous/Heterozygous SNVs in mitochondrial DNA**

| **Reference Allele** | **Variant Allele** | **SNV Location** |
| --- | --- | --- |
| A | T | 1000 |
| A | G | 9001 |
| A | C | 5000 |
| T | A | 2000 |
| T | G | 3000 |
| T | C | 4000 |
| G | A | 7000 |
| G | T | 8001 |
| G | C | 10001 |
| C | A | 6001 |
| C | T | 11000 |
| C | G | 12001 |

**Supplementary Table S2: Location of Homozygous/Heterozygous INDELs in mitochondrial DNA**

| **INDEL Type** | **INDEL Size** | **INDEL Location** |
| --- | --- | --- |
| **Insertion** | 1 | 429 |
|  | 2 | 1730 |
|  | 5 | 2548 |
|  | 10 | 3367 |
| **Deletion** | 1 | 4157 |
|  | 2 | 4784 |
|  | 5 | 5583 |
|  | 10 | 6615 |

**Supplementary Table S3: Performance of SeqVItA in identifying Heterozygous SNVs in simulated data as a function of sequencing depth, read length and minimum coverage threshold. Minimum coverage threshold = 10**

|  |  | **Read Length 100** | | | **Read Length 50** | | |
| --- | --- | --- | --- | --- | --- | --- | --- |
| **Sequencing**  **Coverage** | **Base Quality cut-off** | **Sensitivity** | **Precision** | **F-score** | **Sensitivity** | **Precision** | **F-score** |
| **20x** | **15** | **0.67** | **1** | **0.80** | **0.51** | **1** | **0.68** |
|  | **30** | **0.56** | **1** | **0.72** | **0.04** | **1** | **0.09** |
| **40x** | **15** | **0.98** | **1** | **0.99** | **0.95** | **1** | **0.97** |
|  | **30** | **0.96** | **1** | **0.98** | **0.46** | **1** | **0.63** |
| **60x** | **15** | **1** | **1** | **1** | **0.99** | **1** | **1** |
|  | **30** | **0.99** | **1** | **1** | **0.82** | **1** | **0.90** |

**Supplementary Table S4: Performance of SeqVItA in identifying Homozygous SNVs in simulated data as a function of sequencing depth, read length and minimum coverage threshold. Minimum coverage threshold = 10**

|  |  | **Read Length 100** | | | **Read Length 50** | | |
| --- | --- | --- | --- | --- | --- | --- | --- |
| **Sequencing Coverage** | **Base Quality cut-off** | **Sensitivity** | **Precision** | **F-score** | **Sensitivity** | **Precision** | **F-score** |
| **20x** | **15** | **0.88** | **1** | **0.93** | **0.75** | **1** | **0.86** |
|  | **30** | **0.78** | **1** | **0.87** | **0.06** | **1** | **0.11** |
| **40x** | **15** | **1** | **1** | **1** | **1** | **1** | **1** |
|  | **30** | **0.99** | **1** | **1** | **0.71** | **1** | **0.83** |
| **60x** | **15** | **1** | **1** | **1** | **1** | **1** | **1** |
|  | **30** | **0.99** | **1** | **1** | **0.88** | **1** | **0.94** |

**Supplementary Table S5: Performance of SeqVItA in identifying short homozygous insertions of size 1, 2, 5 and 10 bp in simulated data as a function of sequencing depth, read length and Base quality cut-off. Minimum coverage threshold = 10**

| **Sequencing Coverage** | **Insertion  Size** | **Base Quality**  **cut-off** | **Read Length 100** | | | **Read Length 50** | | |
| --- | --- | --- | --- | --- | --- | --- | --- | --- |
|  |  |  | **Sensitivity** | **Precision** | **F-score** | **Sensitivity** | **Precision** | **F-score** |
| **20x** | **1** | **15** | **0.94** | **1** | **0.97** | **0.78** | **1** | **0.87** |
|  |  | **30** | **0.80** | **1** | **0.88** | **0.06** | **1** | **0.11** |
|  | **2** | **15** | **0.86** | **1** | **0.92** | **0.76** | **1** | **0.86** |
|  |  | **30** | **0.60** | **1** | **0.75** | **0.12** | **1** | **0.21** |
|  | **5** | **15** | **0.76** | **1** | **0.86** | **0.54** | **1** | **0.70** |
|  |  | **30** | **0.66** | **1** | **0.79** | **0.04** | **1** | **0.07** |
|  | **10** | **15** | **0.68** | **1** | **0.80** | **0** | **0** | **0** |
|  |  | **30** | **0.52** | **1** | **0.68** | **0** | **0** | **0** |
| **40x** | **1** | **15** | **1** | **1** | **1** | **1** | **1** | **1** |
|  |  | **30** | **1** | **1** | **1** | **0.74** | **1** | **0.85** |
|  | **2** | **15** | **1** | **1** | **1** | **1** | **1** | **1** |
|  |  | **30** | **1** | **1** | **1** | **0.72** | **1** | **0.84** |
|  | **5** | **15** | **1** | **1** | **1** | **0.98** | **1** | **0.99** |
|  |  | **30** | **1** | **1** | **1** | **0.64** | **1** | **0.78** |
|  | **10** | **15** | **0.96** | **1** | **0.98** | **0** | **0** | **0** |
|  |  | **30** | **0.92** | **1** | **0.95** | **0** | **0** | **0** |
| **60x** | **1** | **15** | **1** | **1** | **1** | **1** | **1** | **1** |
|  |  | **30** | **1** | **1** | **1** | **0.98** | **1** | **0.99** |
|  | **2** | **15** | **1** | **1** | **1** | **1** | **1** | **1** |
|  |  | **30** | **1** | **1** | **1** | **0.98** | **1** | **0.99** |
|  | **5** | **15** | **1** | **1** | **1** | **1** | **1** | **1** |
|  |  | **30** | **1** | **1** | **1** | **0.98** | **1** | **0.99** |
|  | **10** | **15** | **1** | **1** | **1** | **0** | **0** | **0** |
|  |  | **30** | **1** | **1** | **1** | **0** | **0** | **0** |

**Supplementary Table S6: Performance of SeqVItA in identifying short homozygous deletions of size 1, 2, 5 and 10 bp in simulated data as a function of sequencing depth, read length and Base quality. Minimum coverage threshold = 10**

| **Sequencing Coverage** | **Deletion  Size** | **Base Quality**  **cut-off** | **Read Length 100** | | | **Read Length 50** | | |
| --- | --- | --- | --- | --- | --- | --- | --- | --- |
|  |  |  | **Sensitivity** | **Precision** | **F-score** | **Sensitivity** | **Precision** | **F-score** |
| **20x** | **1** | **15** | **0.88** | **1** | **0.94** | **0.84** | **1** | **0.91** |
|  |  | **30** | **0.84** | **1** | **0.91** | **0.06** | **1** | **0.11** |
|  | **2** | **15** | **0.70** | **1** | **0.98** | **0.66** | **1** | **0.79** |
|  |  | **30** | **0.80** | **1** | **0.88** | **0.14** | **1** | **0.24** |
|  | **5** | **15** | **0.70** | **1** | **0.82** | **0.48** | **1** | **0.65** |
|  |  | **30** | **0.54** | **1** | **0.70** | **0** | **0** | **0** |
|  | **10** | **15** | **0.76** | **1** | **0.86** | **0** | **0** | **0** |
|  |  | **30** | **0.62** | **1** | **0.76** | **0** | **0** | **0** |
| **40x** | **1** | **15** | **1** | **1** | **1** | **1** | **1** | **1** |
|  |  | **30** | **1** | **1** | **1** | **0.80** | **1** | **0.88** |
|  | **2** | **15** | **1** | **1** | **1** | **1** | **1** | **1** |
|  |  | **30** | **1** | **1** | **1** | **0.72** | **1** | **0.83** |
|  | **5** | **15** | **1** | **1** | **1** | **1** | **1** | **1** |
|  |  | **30** | **1** | **1** | **1** | **0.54** | **1** | **0.70** |
|  | **10** | **15** | **1** | **1** | **1** | **0** | **0** | **0** |
|  |  | **30** | **1** | **1** | **1** | **0** | **0** | **0** |
| **60x** | **1** | **15** | **1** | **1** | **1** | **1** | **1** | **1** |
|  |  | **30** | **1** | **1** | **1** | **0.98** | **1** | **0.99** |
|  | **2** | **15** | **1** | **1** | **1** | **1** | **1** | **1** |
|  |  | **30** | **1** | **1** | **1** | **0.98** | **1** | **0.99** |
|  | **5** | **15** | **1** | **1** | **1** | **1** | **1** | **1** |
|  |  | **30** | **1** | **1** | **1** | **0.96** | **1** | **0.98** |
|  | **10** | **15** | **1** | **1** | **1** | **0** | **0** | **0** |
|  |  | **30** | **1** | **1** | **1** | **0** | **0** | **0** |

**Supplementary Table S7: Performance of SeqVItA in identifying short heterozygous insertions of size 1, 2, 5 and 10 bp in simulated data as a function of sequencing depth, read length and Base quality cut-off. Minimum coverage threshold = 10**

| **Sequencing Coverage** | **Insertion  Size** | **Base Quality**  **cut-off** | **Read Length 100** | | | **Read Length 50** | | |
| --- | --- | --- | --- | --- | --- | --- | --- | --- |
|  |  |  | **Sensitivity** | **Precision** | **F-score** | **Sensitivity** | **Precision** | **F-score** |
| **20x** | **1** | **15** | **0.56** | **1** | **0.72** | **0.48** | **1** | **0.65** |
|  |  | **30** | **0.40** | **1** | **0.57** | **0.06** | **1** | **0.11** |
|  | **2** | **15** | **0.38** | **1** | **0.55** | **0.24** | **1** | **0.39** |
|  |  | **30** | **0.34** | **1** | **0.51** | **0** | **0** | **0** |
|  | **5** | **15** | **0.28** | **1** | **0.44** | **0.10** | **1** | **0.18** |
|  |  | **30** | **0.22** | **1** | **0.36** | **0** | **0** | **0** |
|  | **10** | **15** | **0.06** | **1** | **0.11** | **0** | **0** | **0** |
|  |  | **30** | **0.04** | **1** | **0.08** | **0** | **0** | **0** |
| **40x** | **1** | **15** | **0.96** | **1** | **0.98** | **0.92** | **1** | **0.96** |
|  |  | **30** | **0.94** | **1** | **0.97** | **0.28** | **1** | **0.44** |
|  | **2** | **15** | **0.86** | **1** | **0.92** | **0.82** | **1** | **0.90** |
|  |  | **30** | **0.84** | **1** | **0.91** | **0.22** | **1** | **0.36** |
|  | **5** | **15** | **0.84** | **1** | **0.91** | **0.56** | **1** | **0.72** |
|  |  | **30** | **0.76** | **1** | **0.86** | **0.14** | **1** | **0.24** |
|  | **10** | **15** | **0.40** | **1** | **0.57** | **0** | **0** | **0** |
|  |  | **30** | **0.36** | **1** | **0.52** | **0** | **0** | **0** |
| **60x** | **1** | **15** | **0.96** | **1** | **0.98** | **1** | **1** | **1** |
|  |  | **30** | **0.96** | **1** | **0.98** | **0.76** | **1** | **0.86** |
|  | **2** | **15** | **0.98** | **1** | **0.99** | **0.94** | **1** | **0.97** |
|  |  | **30** | **1** | **1** | **1** | **0.57** | **1** | **0.73** |
|  | **5** | **15** | **0.92** | **1** | **0.96** | **0.80** | **1** | **0.89** |
|  |  | **30** | **0.90** | **1** | **0.95** | **0.30** | **1** | **0.46** |
|  | **10** | **15** | **1** | **1** | **1** | **0** | **0** | **0** |
|  |  | **30** | **0.56** | **1** | **0.71** | **0** | **0** | **0** |

**Supplementary Table S8: Performance of SeqVItA in identifying short heterozygous deletions of size 1, 2, 5 and 10 bp in simulated data as a function of sequencing depth, read length and Base quality. Minimum coverage threshold = 10**

| **Sequencing Coverage** | **Deletion  Size** | **Base Quality**  **cut-off** | **Read Length 100** | | | **Read Length 50** | | |
| --- | --- | --- | --- | --- | --- | --- | --- | --- |
|  |  |  | **Sensitivity** | **Precision** | **F-score** | **Sensitivity** | **Precision** | **F-score** |
| **20x** | **1** | **15** | **0.56** | **1** | **0.72** | **0.48** | **1** | **0.65** |
|  |  | **30** | **0.46** | **1** | **0.63** | **0.02** | **1** | **0.04** |
|  | **2** | **15** | **0.66** | **1** | **0.80** | **0.30** | **1** | **0.46** |
|  |  | **30** | **0.50** | **1** | **0.67** | **0.04** | **1** | **0.08** |
|  | **5** | **15** | **0.44** | **1** | **0.61** | **0.24** | **1** | **0.39** |
|  |  | **30** | **0.36** | **1** | **0.53** | **0** | **0** | **0** |
|  | **10** | **15** | **0.52** | **1** | **0.68** | **0** | **0** | **0** |
|  |  | **30** | **0.44** | **1** | **0.61** | **0** | **0** | **0** |
| **40x** | **1** | **15** | **0.98** | **1** | **0.99** | **0.92** | **1** | **0.96** |
|  |  | **30** | **0.98** | **1** | **0.99** | **0.48** | **1** | **0.65** |
|  | **2** | **15** | **0.94** | **1** | **0.97** | **0.84** | **1** | **0.91** |
|  |  | **30** | **0.90** | **1** | **0.95** | **0.38** | **1** | **0.55** |
|  | **5** | **15** | **0.98** | **1** | **0.99** | **0.74** | **1** | **0.85** |
|  |  | **30** | **0.94** | **1** | **0.97** | **0.24** | **1** | **0.39** |
|  | **10** | **15** | **0.96** | **1** | **0.98** | **0** | **0** | **0** |
|  |  | **30** | **0.90** | **1** | **0.95** | **0** | **0** | **0** |
| **60x** | **1** | **15** | **1** | **1** | **1** | **0.98** | **1** | **0.99** |
|  |  | **30** | **1** | **1** | **1** | **0.74** | **1** | **0.85** |
|  | **2** | **15** | **1** | **1** | **1** | **0.96** | **1** | **0.98** |
|  |  | **30** | **1** | **1** | **1** | **0.68** | **1** | **0.81** |
|  | **5** | **15** | **1** | **1** | **1** | **0.98** | **1** | **0.99** |
|  |  | **30** | **1** | **1** | **1** | **0.48** | **1** | **0.65** |
|  | **10** | **15** | **1** | **1** | **1** | **0** | **0** | **0** |
|  |  | **30** | **0.96** | **1** | **0.98** | **0** | **0** | **0** |

**Supplementary Table S9: Performance of SeqVItA, BCFtools, VarScan2 and GATK in predicting homozygous SNVs**

| **Method** | **Sequencing depth** | **Recall** | **Precision** | **F-score** |
| --- | --- | --- | --- | --- |
| **SeqVItA** | **20×** | **0.88** | **1.00** | **0.94** |
|  | **40×** | **1.00** | **1.00** | **1.00** |
|  | **60×** | **1.00** | **1.00** | **1.00** |
| **BCFtools** | **20×** | **1.00** | **1.00** | **1.00** |
|  | **40×** | **1.00** | **1.00** | **1.00** |
|  | **60×** | **1.00** | **1.00** | **1.00** |
| **VarScan2** | **20×** | **0.99** | **1.00** | **0.99** |
|  | **40×** | **1.00** | **1.00** | **1.00** |
|  | **60×** | **1.00** | **1.00** | **1.00** |
| **GATK** | **20×** | **0.96** | **1.00** | **0.98** |
|  | **40×** | **0.98** | **1.00** | **0.99** |
|  | **60×** | **1.00** | **1.00** | **1.00** |

**Supplementary Table S10: Performance of SeqVItA, BCFtools, VarScan2 and GATK in predicting heterozygous SNVs**

| **Method** | **Sequencing depth** | **Recall** | **Precision** | **F-score** |
| --- | --- | --- | --- | --- |
| **SeqVItA** | **20×** | **0.67** | **1.00** | **0.80** |
|  | **40×** | **0.98** | **1.00** | **0.99** |
|  | **60×** | **1.00** | **1.00** | **1.00** |
| **BCFtools** | **20×** | **0.94** | **1.00** | **0.97** |
|  | **40×** | **0.98** | **1.00** | **0.99** |
|  | **60×** | **0.98** | **1.00** | **0.99** |
| **VarScan2** | **20×** | **0.69** | **1.00** | **0.82** |
|  | **40×** | **0.99** | **1.00** | **0.99** |
|  | **60×** | **1.00** | **1.00** | **1.00** |
| **GATK** | **20×** | **0.67** | **1.00** | **0.80** |
|  | **40×** | **0.93** | **1.00** | **0.96** |
|  | **60×** | **0.99** | **1.00** | **0.99** |

**Supplementary Table S11: Performance of SeqVItA, BCFtools, VarScan2 and GATK in predicting Homozygous Insertions (1-10 bp)**

| **Method** | **Size** | **20x** | | | **40x** | | | **60x** | | |
| --- | --- | --- | --- | --- | --- | --- | --- | --- | --- | --- |
|  |  | **Recall** | **Precision** | **F-score** | **Recall** | **Precision** | **F-score** | **Recall** | **Precision** | **F-score** |
| **SeqVItA** | **1** | **1.0** | **1.0** | **1.0** | **1.0** | **1.0** | **1.0** | **1.0** | **1.0** | **1.0** |
|  | **2** | **1.0** | **1.0** | **1.0** | **1.0** | **1.0** | **1.0** | **1.0** | **1.0** | **1.0** |
|  | **5** | **0.96** | **1.0** | **0.98** | **1.0** | **1.0** | **1.0** | **1.0** | **1.0** | **1.0** |
|  | **10** | **0.86** | **1.0** | **0.92** | **0.98** | **1.0** | **0.99** | **1.0** | **1.0** | **1.0** |
| **BCFtools** | **1** | **0.94** | **1.0** | **0.97** | **0.96** | **1.0** | **0.98** | **1.0** | **0.96** | **0.98** |
|  | **2** | **1.0** | **1.0** | **1.0** | **1.0** | **1.0** | **1.0** | **1.0** | **0.87** | **0.93** |
|  | **5** | **0.92** | **0.98** | **0.95** | **1.0** | **0.86** | **0.92** | **1.0** | **0.61** | **0.76** |
|  | **10** | **0.0** | **0.0** | **0.0** | **0.0** | **0.0** | **0.0** | **0.0** | **0.0** | **0.0** |
| **VarScan2** | **1** | **0.98** | **1.0** | **0.99** | **1.0** | **1.0** | **1.0** | **1.0** | **1.0** | **1.0** |
|  | **2** | **0.96** | **1.0** | **0.98** | **1.0** | **1.0** | **1.0** | **1.0** | **1.0** | **1.0** |
|  | **5** | **0.32** | **1.0** | **0.48** | **0.80** | **1.0** | **0.89** | **0.96** | **1.0** | **0.98** |
|  | **10** | **0.0** | **0.0** | **0.0** | **0.0** | **0.0** | **0.0** | **0.0** | **0.0** | **0.0** |
| **GATK** | **1** | **0.24** | **1.0** | **0.39** | **0.36** | **1.0** | **0.53** | **0.78** | **0.92** | **0.84** |
|  | **2** | **0.92** | **1.0** | **0.96** | **1.0** | **1.0** | **1.0** | **1.0** | **0.94** | **0.97** |
|  | **5** | **0.70** | **1.0** | **0.82** | **0.96** | **1.0** | **0.98** | **1.0** | **0.98** | **0.99** |
|  | **10** | **0.0** | **0.0** | **0.0** | **0.0** | **0.0** | **0.0** | **0.0** | **0.0** | **0.0** |

**Supplementary Table S12: Performance of SeqVItA, BCFtools, VarScan2 and GATK in predicting Heterozygous Insertions (1-10 bp)**

| **Method** | **Size** | **20x** | | | **40x** | | | **60x** | | |
| --- | --- | --- | --- | --- | --- | --- | --- | --- | --- | --- |
|  |  | **Recall** | **Precision** | **F-score** | **Recall** | **Precision** | **F-score** | **Recall** | **Precision** | **F-score** |
| **SeqVItA** | **1** | **0.56** | **1.0** | **0.72** | **0.96** | **1.0** | **0.98** | **0.96** | **1.0** | **0.98** |
|  | **2** | **0.38** | **1.0** | **0.55** | **0.86** | **1.0** | **0.92** | **0.98** | **1.0** | **0.99** |
|  | **5** | **0.28** | **1.0** | **0.44** | **0.84** | **1.0** | **0.91** | **0.92** | **1.0** | **0.96** |
|  | **10** | **0.06** | **1.0** | **0.11** | **0.40** | **1.0** | **0.57** | **1.0** | **1.0** | **1.0** |
| **BCFtools** | **1** | **0.28** | **1.0** | **0.44** | **0.46** | **1.0** | **0.63** | **0.62** | **1.0** | **0.77** |
|  | **2** | **0.52** | **1.0** | **0.68** | **0.78** | **1.0** | **0.88** | **0.86** | **1.0** | **0.92** |
|  | **5** | **0.28** | **1.0** | **0.44** | **0.66** | **1.0** | **0.80** | **0.62** | **0.94** | **0.75** |
|  | **10** | **0.0** | **0.0** | **0.0** | **0.0** | **0.0** | **0.0** | **0.0** | **0.0** | **0.0** |
| **VarScan2** | **1** | **0.52** | **1.0** | **0.68** | **0.96** | **1.0** | **0.98** | **0.96** | **1.0** | **0.98** |
|  | **2** | **0.34** | **1.0** | **0.51** | **0.88** | **1.0** | **0.94** | **1.0** | **1.0** | **1.0** |
|  | **5** | **0.0** | **0.0** | **0.0** | **0.22** | **1.0** | **0.36** | **0.26** | **1.0** | **0.41** |
|  | **10** | **0.0** | **0.0** | **0.0** | **0.0** | **0.0** | **0.0** | **0.0** | **0.0** | **0.0** |
| **GATK** | **1** | **0.02** | **1.0** | **0.04** | **0.28** | **1.0** | **0.44** | **0.28** | **1.0** | **0.44** |
|  | **2** | **0.57** | **1.0** | **0.73** | **0.94** | **1.0** | **0.97** | **1.0** | **1.0** | **1.0** |
|  | **5** | **0.32** | **1.0** | **0.48** | **0.68** | **1.0** | **0.81** | **0.90** | **1.0** | **0.95** |
|  | **10** | **0.0** | **0.0** | **0.0** | **0.0** | **0.0** | **0.0** | **0.0** | **0.0** | **0.0** |

**Supplementary Table S13: Performance of SeqVItA, BCFtools, VarScan2 and GATK in predicting Homozygous Deletions (1-10 bp)**

| **Method** | **Size** | **20x** | | | **40x** | | | **60x** | | |
| --- | --- | --- | --- | --- | --- | --- | --- | --- | --- | --- |
|  |  | **Recall** | **Precision** | **F-score** | **Recall** | **Precision** | **F-score** | **Recall** | **Precision** | **F-score** |
| **SeqVItA** | **1** | **1.0** | **1.0** | **1.0** | **1.0** | **1.0** | **1.0** | **1.0** | **1.0** | **1.0** |
|  | **2** | **0.98** | **1.0** | **0.99** | **1.0** | **1.0** | **1.0** | **1.0** | **1.0** | **1.0** |
|  | **5** | **0.94** | **1.0** | **0.97** | **1.0** | **1.0** | **1.0** | **1.0** | **1.0** | **1.0** |
|  | **10** | **1.0** | **1.0** | **1.0** | **1.0** | **1.0** | **1.0** | **1.0** | **1.0** | **1.0** |
| **BCFtools** | **1** | **0.76** | **0.97** | **0.85** | **0.96** | **1.0** | **0.98** | **1.0** | **1.0** | **1.0** |
|  | **2** | **1.0** | **0.92** | **0.96** | **1.0** | **0.98** | **0.99** | **1.0** | **1.0** | **1.0** |
|  | **5** | **0.90** | **0.85** | **0.87** | **1.0** | **0.63** | **0.77** | **1.0** | **0.74** | **0.85** |
|  | **10** | **0.0** | **0.0** | **0.0** | **0.0** | **0.0** | **0.0** | **0.0** | **0.0** | **0.0** |
| **VarScan2** | **1** | **0.98** | **1.0** | **0.99** | **1.0** | **1.0** | **1.0** | **1.0** | **1.0** | **1.0** |
|  | **2** | **0.98** | **1.0** | **0.99** | **1.0** | **1.0** | **1.0** | **1.0** | **1.0** | **1.0** |
|  | **5** | **0.48** | **1.0** | **0.65** | **0.70** | **1.0** | **0.82** | **0.72** | **1.0** | **0.84** |
|  | **10** | **0.0** | **0.0** | **0.0** | **0.0** | **0.0** | **0.0** | **0.0** | **0.0** | **0.0** |
| **GATK** | **1** | **0.94** | **1.0** | **0.97** | **1.0** | **1.0** | **1.0** | **1.0** | **1.0** | **1.0** |
|  | **2** | **0.94** | **1.0** | **0.97** | **1.0** | **1.0** | **1.0** | **1.0** | **1.0** | **1.0** |
|  | **5** | **0.14** | **1.0** | **0.25** | **0.38** | **1.0** | **0.55** | **0.57** | **1.0** | **0.73** |
|  | **10** | **0.02** | **1.0** | **0.04** | **0.02** | **1.0** | **0.04** | **0.04** | **1.0** | **0.08** |

**Supplementary Table S14: Performance of SeqVItA, BCFtools, VarScan2 and GATK in predicting Heterozygous Deletions (1-10 bp)**

| **Method** | **Size** | **20x** | | | **40x** | | | **60x** | | |
| --- | --- | --- | --- | --- | --- | --- | --- | --- | --- | --- |
|  |  | **Recall** | **Precision** | **F-score** | **Recall** | **Precision** | **F-score** | **Recall** | **Precision** | **F-score** |
| **SeqVItA** | **1** | **0.56** | **1.0** | **0.72** | **0.98** | **1.0** | **0.99** | **1.0** | **1.0** | **1.0** |
|  | **2** | **0.66** | **1.0** | **0.80** | **0.94** | **1.0** | **0.97** | **1.0** | **1.0** | **1.0** |
|  | **5** | **0.44** | **1.0** | **0.61** | **0.98** | **1.0** | **0.99** | **1.0** | **1.0** | **1.0** |
|  | **10** | **0.52** | **1.0** | **0.68** | **0.96** | **1.0** | **0.98** | **1.0** | **1.0** | **1.0** |
| **BCFtools** | **1** | **0.08** | **1.0** | **0.15** | **0.16** | **1.0** | **0.28** | **0.24** | **1.0** | **0.39** |
|  | **2** | **0.48** | **1.0** | **0.65** | **0.62** | **1.0** | **0.77** | **0.88** | **0.96** | **0.92** |
|  | **5** | **0.22** | **1.0** | **0.36** | **0.38** | **1.0** | **0.54** | **0.46** | **1.0** | **0.63** |
|  | **10** | **0.0** | **0.0** | **0.0** | **0.0** | **0.0** | **0.0** | **0.0** | **0.0** | **0.0** |
| **VarScan2** | **1** | **0.54** | **1.0** | **0.70** | **0.98** | **1.0** | **0.99** | **1.0** | **1.0** | **1.0** |
|  | **2** | **0.66** | **1.0** | **0.80** | **0.94** | **1.0** | **0.97** | **1.0** | **1.0** | **1.0** |
|  | **5** | **0.02** | **1.0** | **0.04** | **0.24** | **0.95** | **0.37** | **0.50** | **1.0** | **0.61** |
|  | **10** | **0.0** | **0.0** | **0.0** | **0.0** | **0.0** | **0.0** | **0.0** | **0.0** | **0.0** |
| **GATK** | **1** | **0.52** | **1.0** | **0.68** | **0.88** | **1.0** | **0.94** | **0.98** | **1.0** | **0.99** |
|  | **2** | **0.64** | **1.0** | **0.78** | **0.96** | **1.0** | **0.98** | **0.98** | **1.0** | **0.99** |
|  | **5** | **0.0** | **0.0** | **0.0** | **0.08** | **1.0** | **0.15** | **0.28** | **1.0** | **0.44** |
|  | **10** | **0.0** | **0.0** | **0.0** | **0.0** | **0.0** | **0.0** | **0.0** | **0.0** | **0.0** |

**Supplementary Table S15: Distribution of Sequence variants in 24 Hepatocellular Carcinoma Patients**

| **Patient ID** | **SNVs** | | | | **Indels** | | | |
| --- | --- | --- | --- | --- | --- | --- | --- | --- |
|  | **Germline** | **Somatic** | **LOH** | **Unknown** | **Germline** | **Somatic** | **LOH** | **Unknown** |
| **1** | 1612 | 60 | 24 | 0 | 91 | 5 | 3 | 0 |
| **2** | 2284 | 64 | 113 | 2 | 224 | 8 | 23 | 1 |
| **4** | 1489 | 39 | 5 | 0 | 97 | 2 | 3 | 1 |
| **5** | 1507 | 64 | 76 | 0 | 91 | 8 | 6 | 0 |
| **6** | 1883 | 35 | 172 | 0 | 209 | 15 | 26 | 0 |
| **7** | 1337 | 75 | 78 | 0 | 84 | 6 | 11 | 0 |
| **8** | 2313 | 47 | 132 | 0 | 224 | 9 | 18 | 2 |
| **9** | 1378 | 45 | 78 | 0 | 97 | 7 | 6 | 3 |
| **10** | 2509 | 91 | 235 | 2 | 237 | 12 | 31 | 1 |
| **11** | 1467 | 74 | 73 | 1 | 96 | 4 | 10 | 0 |
| **12** | 1497 | 59 | 31 | 1 | 101 | 10 | 8 | 0 |
| **13** | 2494 | 45 | 27 | 1 | 258 | 6 | 5 | 1 |
| **14** | 1967 | 70 | 181 | 31 | 144 | 4 | 66 | 16 |
| **15** | 2198 | 26 | 41 | 0 | 233 | 3 | 7 | 0 |
| **16** | 2356 | 37 | 24 | 2 | 256 | 8 | 5 | 1 |
| **17** | 2349 | 28 | 25 | 0 | 228 | 6 | 4 | 0 |
| **18** | 1475 | 51 | 34 | 1 | 96 | 8 | 5 | 0 |
| **19** | 1545 | 114 | 62 | 0 | 108 | 10 | 1 | 1 |
| **20** | 1514 | 48 | 30 | 0 | 99 | 4 | 1 | 0 |
| **21** | 1492 | 47 | 56 | 1 | 101 | 12 | 4 | 0 |
| **22** | 1995 | 111 | 252 | 1 | 208 | 15 | 32 | 0 |
| **23** | 2425 | 58 | 35 | 0 | 248 | 10 | 13 | 0 |
| **24** | 2326 | 50 | 19 | 0 | 246 | 4 | 3 | 1 |
| **26** | 1563 | 707 | 623 | 124 | 169 | 80 | 84 | 11 |

**Supplementary Table S16: List of recurrent Somatic Mutated Genes, mutations and their locations along with Patient IDs**

| **Gene** | **Location** | **Mutation** | **Patient IDs** | **Database** |
| --- | --- | --- | --- | --- |
| TP53 | chr17:7577427 (intron) | G>A | 1, 5 | - |
|  | chr17:7578212(coding, frameshift) | G[AA/--] | 1 | COSMIC |
|  | chr17:7578231(coding, frameshift) | C[A/-] | 2 | COSMIC |
|  | chr17:7577407 (intron) | A>C | 5 | - |
|  | chr17:7578427(coding) | T>A | 6 | COSMIC |
|  | chr17:7577534(coding) | C>A | 7,12 | COSMIC, OMIM |
|  | chr17:7577046(coding) | C>A | 9 | COSMIC |
|  | chr17:7578208(coding) | T>C | 10 | COSMIC |
|  | chr17:7579521(coding) | C>A | 18 | COSMIC |
|  | chr17:7578463(coding) | C>G | 24 | - |
| FGFR1 | chr8:38282294(intron) | C>T | 1,7,11,24 | - |
|  | chr8:38272542(intron) | C>G | 1,7,13,21 | - |
|  | chr8:3828755(intron) | C>T | 1 | - |
|  | chr8:38272582(intron) | C>A | 13,23 | - |
|  | chr8:38287555(intron) | C>T | 13 | - |
| FANCD2 | chr3:10089549(intron, frameshift) | T[ACTA/-] | 2,8 | - |
|  | chr3:10088266(coding) | G>T | 7,9 | ClinVar, COSMIC |
|  | chr3:10085624(intron) | G>T | 15 | - |
|  | chr3:10088404(coding) | C>T | 20 | ClinVar, COSMIC |
|  | chr3:10091035(intron) | T>C | 21 | ClinVar |
| MIR1278 | chr1:193104827(upstream) | G>A | 1,6,7,9,10,17,21 | - |
| JAK1 | chr1:65307409(intron) | G>A | 2,9,11 | - |
|  | chr1:65301669(intron) | C>A | 5,8 | - |
|  | chr1:65301317(intron) | G>A | 8 | - |
|  | chr1:65301611(intron) | T>G | 23 | - |
|  | chr1:65312994(intron) | A>G | 23 | - |
| NCOR1 | chr17:15968143(intron) | T>C | 1 | - |
|  | chr17:16097870(coding) | C>A | 1 | COSMIC |
|  | chr17:16068362(coding) | C>T | 2 | COSMIC |
|  | chr17:16068377(coding) | C>G | 2 | COSMIC |
|  | chr17:16068383(coding) | G>A | 16 | COSMIC |
|  | chr17:15961993(intron) | T>A | 23 | - |
|  | chr17:15943910(intron) | T>C | 24 | - |
|  | chr17:15977043(Intron) | C>T | 24 | - |
|  | chr17:16041306(intron) | C>A | 24 |  |
| NUP93 | chr16:56871480(intron) | A>C | 2,23 | - |
|  | chr16:56873613(coding, frameshift) | T[-/A] | 6,16 | - |
|  | chr16:56871481(intron) | C[-/T] | 23 | - |
|  | chr16:56868604(intron) | A>T | 24 | - |
| XPO1 | chr2:61713209(intron) | G[GAAAATC/-] | 2,5,9,11,17 | - |
|  | chr2:61713218(intron) | T>A | 5,11,17 | - |
|  | chr2:61713220(intron) | T>G | 5,11,17 | - |
| SDHC | chr1:161332346(3 UTR) | C>G | 7,12,18,20 | ClinVar |
|  | chr1:161310519(intron) | C>T | 7,12,20 | - |
|  | chr1:161293496(intron) | T>A | 9 | ClinVar |
| TSC2 | chr16:2110571(intron) | C>G | 7,18 | - |
|  | chr16:2120402(intron) | T>C | 11 | - |
|  | chr16:2098680(coding) | G>T | 13 | - |
|  | chr16:2111790(intron) | G>A | 23 | - |

**Supplementary Table S17: Functional annotations of somatic sequence variants identified by SeqVItA in liver cancer patient 9, 19 and 22 (MS Excel file)**

1. **Supplementary Figures**


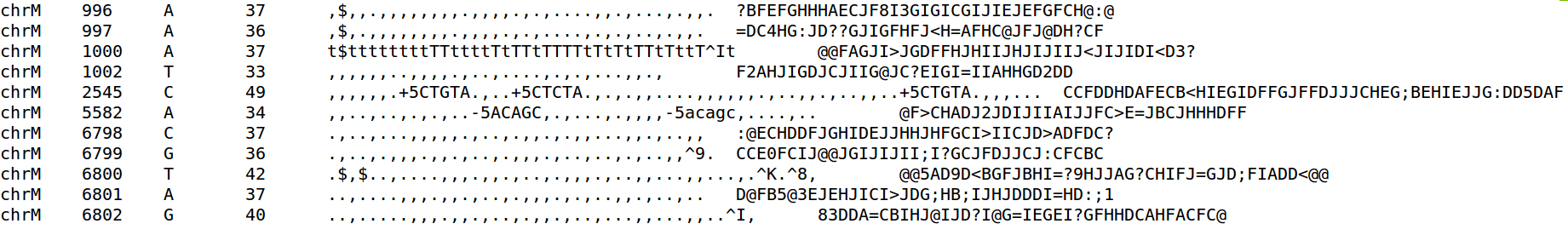


**Supplementary Figure S1: A snapshot of mpileup format generated for a single sample by Samtools**


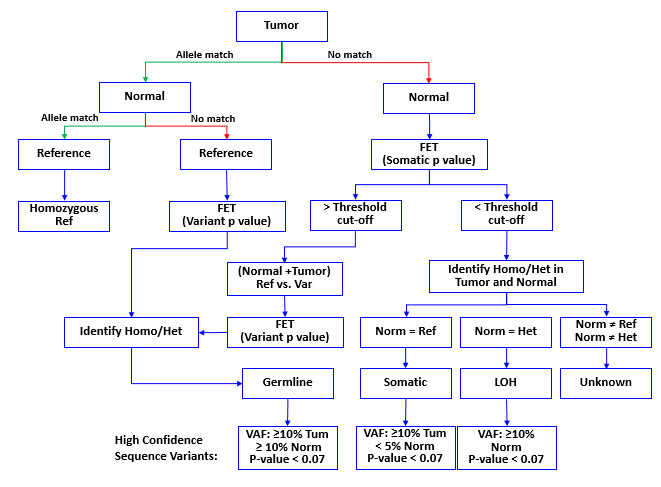


**Supplementary Figure S2: Various criteria considered in the *somatic* module of SeqVItA (Ref: Reference allele, Var: Variant allele, FET: Fisher Exact Test, Norm: Normal, Tum: Tumor, Homo: Homozygous, Het: Heterozygous, LOH: Loss of Heterozygosity)**


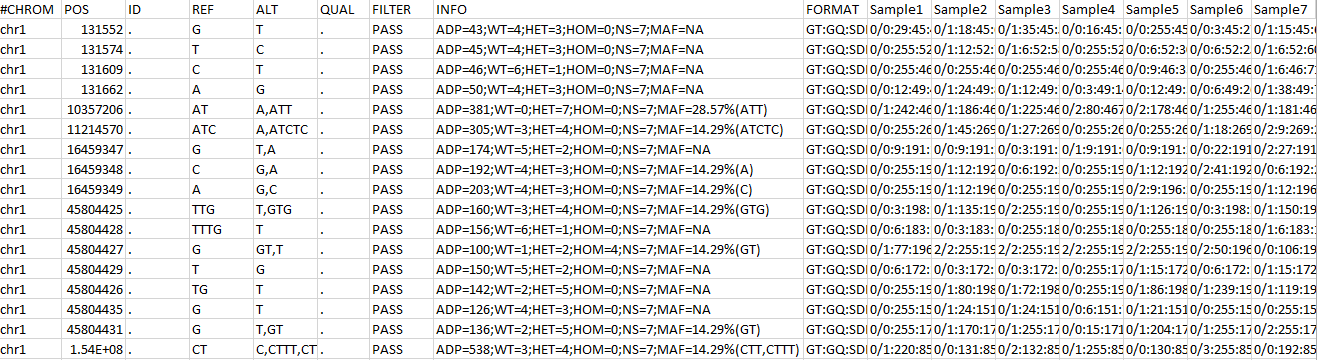


**Supplementary Figure S3: Snapshot of SeqVItA output file on using *population* module across multiple data samples**


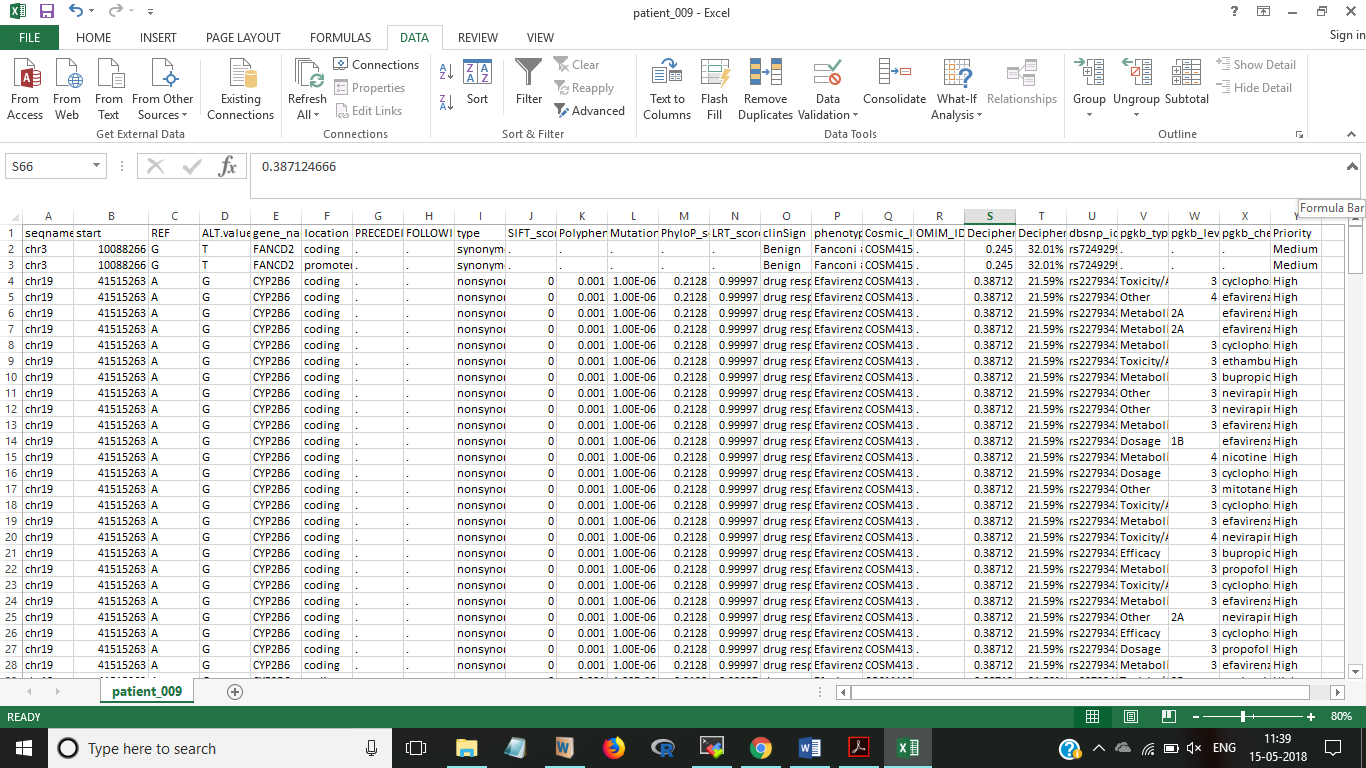


**Supplementary Figure S4: Snapshot of annotation file representing various annotations generated using SeqVItA**


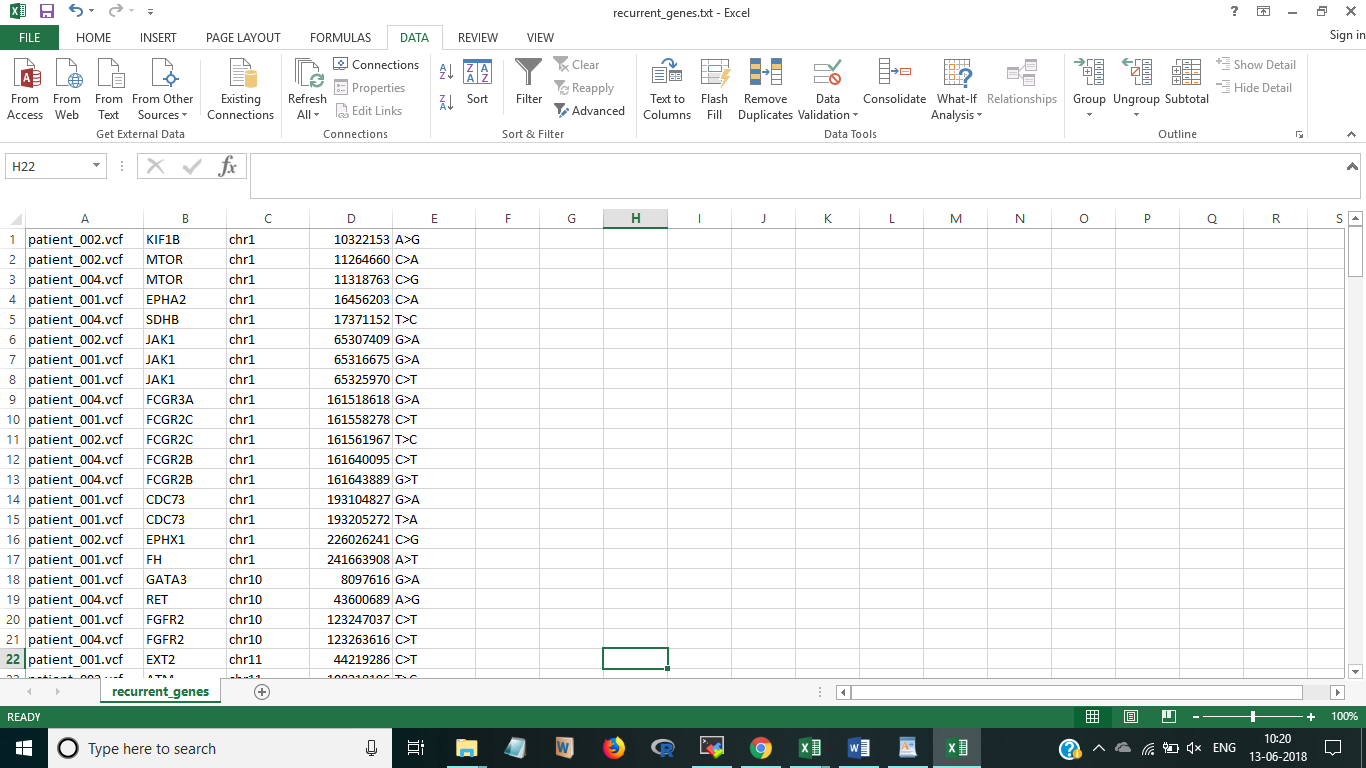


**Supplementary Figure S5: Snapshot showing the functionality of *findRecurrentGenes*. List of samples (patient_001.vcf, patient_002, and patient_004.vcf in this case), genes spanning sequence variants and positions of sequence variants are generated.**


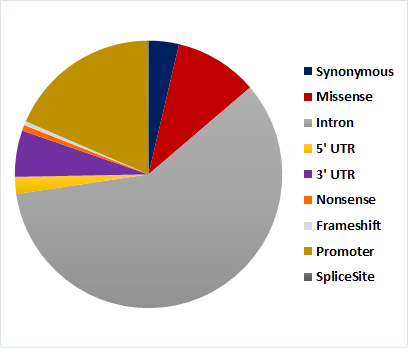


**Figure S6: Distribution of somatic sequence variants predicted across the 24 HCC patient samples (Synonymous, Missense, and Nonsense are mutations in the coding region)**
